# Supplementary figures and images for: Sociological variety and the transmission efficiency of Mycobacterium tuberculosis: a secondary analysis of qualitative and quantitative data from 15 communities in Zambia
Source: BMJ Open. 2021 Dec 14;11(12):e047136. doi: 10.1136/bmjopen-2020-047136 (PMC8671921; doi:10.1136/bmjopen-2020-047136)

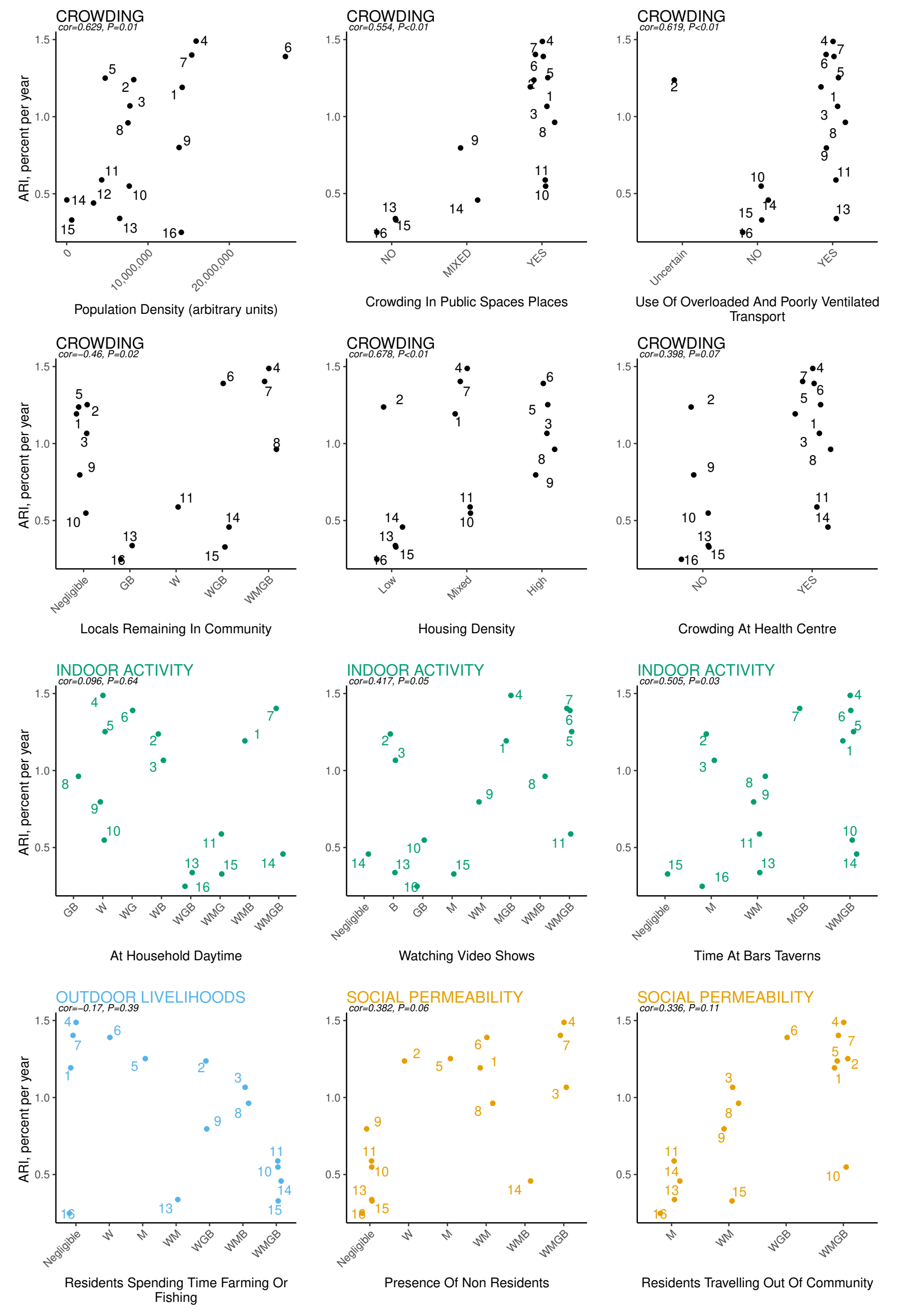

Supplement: Supplementary data [file bmjopen-2020-047136supp001.pdf]

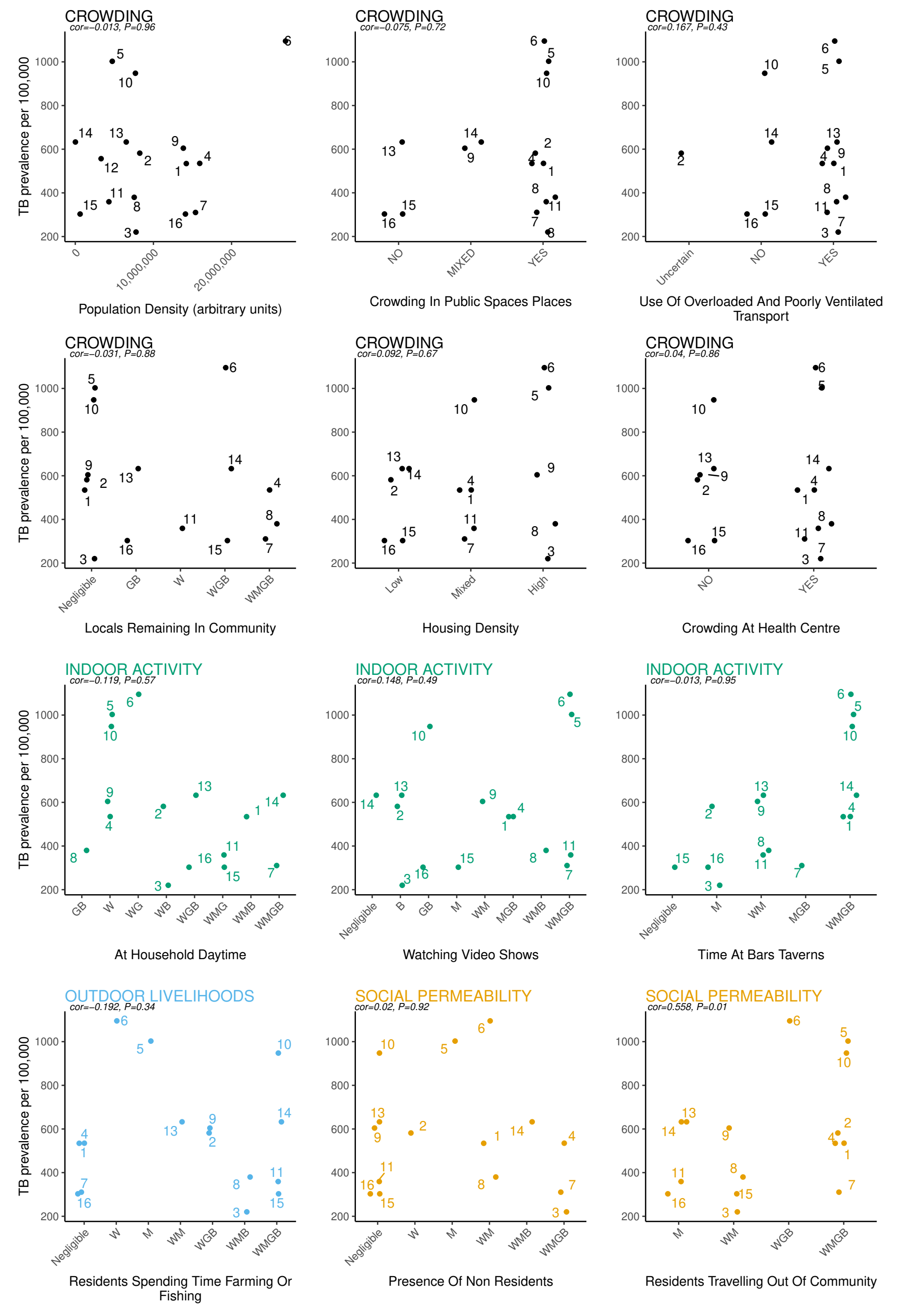

Supplement: Supplementary data [file bmjopen-2020-047136supp002.pdf]
