# Supplementary material for: Sociological variety and the transmission efficiency of Mycobacterium tuberculosis: a secondary analysis of qualitative and quantitative data from 15 communities in Zambia
Source: BMJ Open. 2021 Dec 14;11(12):e047136. doi: 10.1136/bmjopen-2020-047136 (PMC8671921; doi:10.1136/bmjopen-2020-047136)
Supplement: Author's manuscript [file bmjopen-2020-047136.draft_revisions.pdf]

BMJ Open is committed to open peer review. As part of this commitment we make the peer review history of every article we publish publicly available.

When an article is published we post the peer reviewers' comments and the authors' responses online. We also post the versions of the paper that were used during peer review. These are the versions that the peer review comments apply to.

The versions of the paper that follow are the versions that were submitted during the peer review process. They are not the versions of record or the final published versions. They should not be cited or distributed as the published version of this manuscript.

BMJ Open is an open access journal and the full, final, typeset and author-corrected version of record of the manuscript is available on our site with no access controls, subscription charges or pay-per-view fees (http://bmjopen.bmj.com).

If you have any questions on BMJ Open's open peer review process please email info.bmjopen@bmj.com

Sociological Variety and the Transmission Efficiency of Mycobacterium Tuberculosis: A Secondary Analysis of Qualitative and Quantitative Data from Fifteen Communities in Zambia.

Journal:	*BMJ Open*
Manuscript ID	bmjopen-2020-047136
Article Type:	Original research
Date Submitted by the Author:	19-Nov-2020

Complete List of Authors:	Murray, Emma; Previously affiliated with The Desmond Tutu TB Centre, Stellenbosch Univeristy<br>Dodd, PJ; The University of Sheffield, School of Health and Related Research<br>Marais, B; The University of Sydney, Marie Bashier Institute and the Discipline of Child and Adolescent Health<br>Ayles, Helen; London School of Hygiene & Tropical Medicine; Zambart, School of Public Health, University of Zambia<br>Shanaube, Kwame; Zambart, School of Public Health, University of Zambia<br>Schaap, Albertus; London School of Hygiene & Tropical Medicine, Department of Infectious Disease Epidemiology<br>White, RG; London School of Hygiene & Tropical Medicine, Department of Infectious Disease Epidemiology<br>Bond, Virginia; London School of Hygiene & Tropical Medicine, Global Health and Development ; Zambart, School of Public Health, University of Zambia

Keywords:	Tuberculosis < INFECTIOUS DISEASES, SOCIAL MEDICINE, QUALITATIVE RESEARCH, PUBLIC HEALTH, Tropical medicine < INFECTIOUS DISEASES

SCHOLARONE™
Manuscripts

I, the Submitting Author has the right to grant and does grant on behalf of all authors of the Work (as defined in the below author licence), an exclusive licence and/or a non-exclusive licence for contributions from authors who are: i) UK Crown employees; ii) where BMJ has agreed a CC-BY licence shall apply, and/or iii) in accordance with the terms applicable for US Federal Government officers or employees acting as part of their official duties; on a worldwide, perpetual, irrevocable, royalty-free basis to BMJ Publishing Group Ltd ("BMJ") its licensees and where the relevant Journal is co-owned by BMJ to the co-owners of the Journal, to publish the Work in this journal and any other BMJ products and to exploit all rights, as set out in our licence.

The Submitting Author accepts and understands that any supply made under these terms is made by BMJ to the Submitting Author unless you are acting as an employee on behalf of your employer or a postgraduate student of an affiliated institution which is paying any applicable article publishing charge ("APC") for Open Access articles. Where the Submitting Author wishes to make the Work available on an Open Access basis (and intends to pay the relevant APC), the terms of reuse of such Open Access shall be governed by a Creative Commons licence – details of these licences and which Creative Commons licence will apply to this Work are set out in our licence referred to above.

Other than as permitted in any relevant BMJ Author's Self Archiving Policies, I confirm this Work has not been accepted for publication elsewhere, is not being considered for publication elsewhere and does not duplicate material already published. I confirm all authors consent to publication of this Work and authorise the granting of this licence.

For peer review only

Sociological Variety and the Transmission Efficiency of Mycobacterium Tuberculosis: A Secondary Analysis of Qualitative and Quantitative Data from Fifteen Communities in Zambia.

Emma J Murray[1], Peter J Dodd[2], Ben Marais[3], Helen Ayles[4,5], Kwame Shanaube[4], Albertus Schaap[6], Richard White[6], Virginia Bond[4,5]

1. Desmond Tutu TB Centre, Stellenbosch University, Tygerberg, Cape Town, South Africa (previously).
2. School of Health and Related Research, University of Sheffield, Sheffield, United Kingdom.
3. Clinical School, Children's Hospital at Westmead, University of Sydney, Sydney, Australia.
4. Zambart, School of Public Health, University of Zambia, Lusaka, Zambia.
5. Department of Global Health and Development, Faculty of Public Health and Policy, London School of Hygiene & Tropical Medicine, United Kingdom.
6. TB Modelling Group, Department of Infectious Disease Epidemiology, London School of Hygiene and Tropical Medicine, United Kingdom.

For peer review only

Abstract: 299 words

Main Text: (excluding title page, abstract, summary, references, figures and table): 3999 words

Number of References: 32

Table 1: Relational Categorisation of Socio-Spatial Tendencies across Communities

Figure 1: Transmission Efficiency (ratio of ARI to Disease Prevalence) by Socio-Spatial Variables

Figure S1 (supplemental material)**:** Annual Risk of Infection by Socio-Spatial Variables

Figure S2 (supplemental material): Prevalence of Disease by Socio-Spatial Variables (supplemental material)

Corresponding Author:

Emma Jane Murray
83 Highsett
Cambridge
CB2 1NZ
United Kingdom

emmajane.murray@gmail.com

ABSTRACT

Background: High Tuberculosis (TB) burden communities in Zambia were selected as part of a clinical trial named the Zambia and South Africa TB and AIDS Reduction Study (ZAMSTAR). There was wide variability in the prevalence of *Mycobacterium Tuberculosis* (*Mtb*) infection and TB disease across these trial communities.

Objectives: To clarify whether certain communities could have been more (or less) vulnerable to *Mtb* transmission as a result of sociological variety. We aimed to identify and synthesise sociological variables, judged relevant to socio-spatial aspects of *Mtb* transmission, investigating relevance for transmission efficiency.

Design: A mixed methods secondary analysis was conducted using existing ZAMSTAR data. Firstly, we analysed qualitative data to categorise patterns of social and spatial engagement across communities, assessing sociological differences. Secondly, we compared emergent sociological variables with a measure of transmission efficiency: the ratio of annual risk of infection to TB prevalence in a particular community.

Setting: Fifteen communities across Zambia, sharing high burdens of TB and human immunodeficiency virus (HIV), as selected for ZAMSTAR.

Results: Crowding, social permeability, livelihoods and participation in recreational activities differed across communities, based on twelve indicators. Communities were qualitatively classified as more/ less spatially crowded and as more/less socially 'open' to contact with 'outsiders' and places, with implications for the presumptive risk of *Mtb* transmission. For example, watching video shows in poorly ventilated structures posed a presumptive risk in more socially 'open' communities, while outdoor farming and/or fishing were widespread in communities with lower transmission measures.

Conclusion: In the Zambian trial communities, a dual dynamic of social permeability and spatial crowding appeared relevant to *Mtb* transmission efficiency. Certain socio-spatial aspects carrying transmission risk could be adjusted e.g. increasing ventilation on transport. However, other more structural aspects are less malleable e.g. reliance on public transport. Socio-spatial variation may help explain disparities in *Mtb* transmission.

Key Words: tuberculosis transmission, community context, interdisciplinary study

For peer review only

SUMMARY

Strengths and Limitations of this study:

- We used mixed-methods to analyse communities showing disparities in *Mtb* infection and TB disease prevalence.
- Qualitative data successfully captured observed and overt aspects of community life, particularly relevant to *Mtb* transmission.
- Qualitative findings were relationally categorised in order to merge with respective quantitative measures of 'transmission efficiency' across communities.
- Findings generated a grounded and sociologically informed hypothesis that offers to help explain the clinical variability measured by the ZAMSTAR trial.
- Although the study is exploratory, this limitation is offset by the opportunity for such an unusual interdisciplinary endeavour, our applied approach and the significance of socio-spatial context for *Mtb* transmission.

Data Sharing Statement: Qualitative data are available from the last author upon reasonable request. Quantitative ZAMSTAR data are available from the sixth author upon reasonable request. Extra CODA study data are at Zenodo. DOI: http://doi.org/10.5281/zenodo.3874675. Relevant license details have Creative Commons Attribution (https://creativecommons.org/licenses/by/4.0/legalcode)

INTRODUCTION

Interactions between the tuberculosis (TB) and human immunodeficiency virus (HIV) epidemics have complicated and compromised TB containment efforts in Zambia and other high burden countries. There is evidence that as the HIV epidemic ages, an increasing burden of undiagnosed TB is emerging amongst older adults in Zambia, underscoring the resilience of epidemic TB in the region. [1 – 5] Understanding the role of socio-economic factors in facilitating the transmission of *Mycobacterium Tuberculosis* (*Mtb)*, within and outside households, is critical to developing alternative approaches that could reduce ongoing *Mtb* transmission at the community level.[6-11] This inquiry explores how socio-spatial aspects of every-day community life might be relevant to differing *Mtb* transmission dynamics. We used a novel mixed-methods interdisciplinary approach to conduct a secondary analysis of Zambian data, collected as part of a community randomised trial (CRT) - the Zambia and South Africa TB and AIDS Reduction Study (ZAMSTAR).[12] The approach combined community level qualitative data, focused on the use of space and daily social interactions, with quantitative

For peer review only

measurements of TB prevalence and annual risk of infection (ARI), to investigate whether differences in socio-spatial aspects across communities corresponded to a higher or lower *Mtb* transmission efficiency (measured as the ARI per prevalent TB case in a community).

ZAMSTAR (2004-2011) compared two interventions to reduce *Mtb* transmission and TB prevalence across Zambia and South Africa at a population level. The two interventions - enhanced community TB case-finding and household level combined TB-HIV care - were implemented from 2006 to 2009. In Zambia, sixteen communities were selected by TB notification rates in excess of 400/100,000 per annum and a high HIV prevalence.[12-15] Baseline prevalence of *Mtb* infection, as measured by tuberculin skin test (TST) surveys in primary school children, was highly variable, ranging from two to thirteen percent across the Zambian communities and allowed for the estimation of ARI.[13] The final ZAMSTAR prevalence survey found large variation in culture-confirmed TB prevalence. This variability implied, in part, differences in *Mtb* transmission dynamics. Towards the end of ZAMSTAR, an ancillary study, named the Contact Observations of Daily Activities (CODA) Study, sought to measure patterns of social contacts relevant to *Mtb* transmission and model the implied ARIs in adult age groups.[16] Our inquiry supplements the CODA quantitative analysis of adult social contacts, investigating social and spatial contextual variables that may influence community level *Mtb* transmission.

METHODS

This secondary analysis drew on four data sources: qualitative data collected for the purpose of the CODA study; estimates of population density from the ZAMSTAR household enumeration; estimates of ARI from ZAMSTAR TST surveys; and TB prevalence data from final ZAMSTAR TB prevalence survey. Details of ZAMSTAR household enumeration, TST data and implied ARI estimates, TB prevalence data and linked methods have been published.[12, 13, 15] We focus on the CODA qualitative data collection and analysis used for the identification of socio-spatial variables; our use of quantitative ARI and prevalence data from ZAMSTAR; and the mixed method analysis approach that investigated relationships between socio-spatial variables and calculated *Mtb* 'transmission efficiency'.

Qualitative Data

Following the intervention period of ZAMSTAR, Broad Brush Surveys (BBS) were conducted in 2011 across Zambian ZAMSTAR communities as part of CODA.[17] The BBS aimed to a) update previous BBS data collected as part of ZAMSTAR between 2004 and 2005,[9, 18, 19], and b) to provide insight into patterns of behaviour and settings of adult-to-child social mixing, in order to inform the design of a quantitative questionnaire.[16] The BBS fieldwork was completed by thirty-two research assistants

For peer review only

trained over a two-day period in participatory methods. Fieldwork was conducted by a team of two (usually one man and one woman) over a period of five days in each community, using local languages. In sequence, each pair carried out a transect walk through the hub of the community; structured observations of different gender and age spaces (older men/women, younger men/women, boys/girls); structured observation of the health facility, church services and the main transport depot; and day long household observations (two per community). The focus of these observations were presence and contact between adults and children. Daily activity charts were drawn up with boys and girls during the observations, in their spaces. Each team documented observations and conversations in the form of a written 'activity report'. Activity reports were semi-structured, open-ended and written in English, with translation completed in the process of writing by the relevant fieldworkers. Research teams were debriefed on a daily basis, both within the pair of research assistants and remotely over the phone. The second and last authors were part of the training and fieldwork.

The qualitative BBS data indicated how places and transport were used by different age groups and genders. For the purposes of this analysis, we identified and categorised patterns of social and spatial engagement using conversational and observational material from the BBS activity reports, until a point of saturation was reached for each community. This was done in frequent consultation with a key member of the fieldwork team, who also guided the first author in conducting a relational comparison of the patterns identified across communities. One of the sixteen communities was dropped from our analysis due to poor data quality. Similarities and differences were subsequently compared across the remaining fifteen communities. We configured findings into an annotated tabular format of socio-spatial tendencies, broken down across age and gender categories where appropriate (Table 1).

For peer review only

TABLE 1: Relational Categorisation of Socio-Spatial Tendencies across Communities

Community ID Number	1	2	3	4	5	6	7	8	9	10	11	13	14	15	16
INDICATORS OF CROWDING:															
General Crowding in Public Spaces/ Places	YES	YES	YES	YES	YES	YES	YES	YES	Mixed	YES	YES	NO	Mixed	NO	NO
Use of Overloaded and Poorly Ventilated Transport	YES	Uncertain	YES	YES	YES	YES	YES	YES	YES	NO	YES	YES	NO	NO	NO
Locals Remaining in Community (daytime)	Negligible	Negligible	Negligible	M W B G	Negligible	W G B	M W G B	M W B G	Negligible	Negligible	W	B G	W G B	W G B	B G
Housing Density	Mixed	Low	High	Mixed	High	High	Mixed	High	High	Mixed	Mixed	Low	Low	Low	Low
Crowding at Health Centre	YES	NO	YES	YES	YES	YES	YES	YES	NO	NO	YES	NO	YES	NO	NO
Crowding at Churches	YES	YES	NO	YES	YES	YES	YES	YES	YES	YES	NO	YES	NO	NO	NO
PARTICIPATION IN INDOOR ACTIVITIES:															
At Household (daytime)	M W B	W B	W B	W	W	W G	M W B G	B G	W	W	M W G	W B G	M W B G	M W G	W B G
Watching Video Shows	M G B	B	B	M G B	M W B G	M W B G	M W B G	M W B	WM	GB	M W B G	B	Negligible	M	GB
Time at Bars/Taverns	M W B G	M	M	M W B G	M W B G	M W B G	M G B	M W	M W	M W B G	M W	M W	M W B G	Negligible	M
To Church	M W B G	M W B G	M W B G	M W B G	M W B G	M W B G	M W B G	M W B G	M W B G	M W B G	M W B G	M W B G	M W B G	M W B G	M W B G
INDICATORS OF ECONOMY:															
Residents Farming / Fishing	Negligible	W G B	W M B	Negligible	M	W	Negligible	W M B	W G B	M W B G	M W B G	W M	M W B G	M W B G	Negligible
Residents at local market	M W	M	Negligible	M G B	W B	M G B	W G B	M W	W	W	W G	W	Negligible	M W	W
Residents using Local Shops/ Stalls	M	Negligible	M W	M G B	M	M W	M	M	Negligible	M	Negligible	M	M	M	M
INDICATORS OF SOCIAL PERMEABILITY:															
Presence of Non-Residents	M W	W	M W B G	M W B G	M	M W	M W B G	M W	Negligible	Negligible	Negligible	Negligible	W M B	Negligible	Negligible
Residents Travelling Out of Community	M W B G	M W B G	M W	M W B G	M W B G	M W B G	M W B G	M W	M W	M W B G	M	M	M	M W	M
Residents Travelling to External Market	M W	Negligible	W	M W	W	M W	M W	Negligible	W	W	Negligible	W	Negligible	W	W
Social Mixing (general) across Age and Gender	Some	YES	YES	YES	YES	Some	Some	YES	Some	YES	Least	Some	YES	Less	Least
At Health Centre	M W B G	Negligible	M W B G	W G B	M W B G	M W B G	W G B	M W B G	W	W	M W B G	W G B	W G B	W	W
OTHER:															
Children Not Attending School	Negligible	Negligible	Negligible	B G	B G	Negligible	B G	Negligible	Negligible	Negligible	Negligible	Negligible	Negligible	Negligible	B G
To Salon/Barber	W	Negligible	Negligible	W	Negligible	M W G	W	Negligible	W	W	W	Negligible	W	W	W
To Outdoor Water Point	W G	Negligible	Negligible	W G	W	Negligible	W G B	W	Negligible	W G	Negligible	G	Negligible	Negligible	W
To Outdoor Recreation	B	B	M	M B G	M B	Negligible	M B G	B	Negligible	B	B G	B	M B	M B	B G

LEGEND:

Rows of variables were identified and thematically grouped following qualitative BBS data analysis. Themes are capitalised.

Each column provides a ‘snapshot’ of general tendencies within a particular community. Qualitative assessments of variables were relationally gauged across communities, using BBS data.

M = Men

W = Women

B = Boys

G = Girls

For peer review only

We delineated the strongest variables as broadly linked to one or more of the following aspects: 1) levels of spatial crowding, pertaining to the amount of people within shared/confined spaces and the density of physical structures; 2) the range and degree of participation in predominantly indoor recreational activities; 3) the dominance of outdoor livelihoods e.g. widespread commitment to farming or fishing dramatically shaped socio-spatial engagement in certain communities; and 4) what we have termed as 'social permeability' i.e. the level of exchange between local residents and outsiders across and within geographical boundaries, with some communities clearly more socially 'open' to exchange across their geographical boundaries. Eleven qualitative indicators were identified to represent assessments of socio-spatial variability, selected on the basis of being observable (tangible) and categorical, with perceived relevance for TB transmission. The indicators were: general crowding in public spaces/places; the use of overloaded and poorly ventilated transport; local residents remaining in their residential community during the day; housing density; crowding at health centres; time spent at home during the day; watching video shows; going to bars/taverns; spending time farming/ fishing; the presence of non-residents; and evidence of residents travelling out of their residential community.

Quantitative Analysis

As part of ZAMSTAR, TST surveys were conducted in a cohort of primary school children to calculate ARI across communities.[15] For our mixed method analysis, we calculated the ARI-to-TB prevalence ratio, which is an established measure of 'transmission efficiency' (beta, in infectious disease modelling) and likely to be influenced by social features that affect the patterns and intensity of mixing relevant to *Mtb* transmission. Karel Styblo used this ratio to help describe historical patterns in European TB epidemiology. While the values that Styblo found no longer apply, the ratio remains a useful metric.[20] We calculated this ratio for each community using calculated ARI point estimates and TB prevalence point estimates determined during ZAMSTAR. We also computed Kendall Rank correlation coefficients (Spearman correlation coefficient for the quantitative indicator, population density) and approximate P-values. Given the number of variables considered, it is not appropriate to consider P-values below a certain threshold as 'significant'; they simply provide some guidance on how likely it is an apparent individual pattern could arise by chance.

Mixed Methods Analysis

After completing the qualitative analysis and quantitative calculations described above, we first correlated the measure of transmission efficiency with the eleven qualitative variables. Each variable was titled according to one of the four social aspects to which it most strongly indicated. For example,

For peer review only

the variable 'Presence of Non-Residents' was regarded as a strong indicator of social permeability, given the manner in which it presented in the BBS. Qualitative variables (indicators) were also ranked by a priori expectations of increasing transmission risk. For example, "high" > "low" for housing density, or "WMGB" > "M" for women, men, girls and boys spending time in bars versus only men. Expectations of the burden of infectious tuberculosis led to men's involvement being ranked higher than women's involvement, and adults' involvement higher than children's. We also included an objective quantitative indicator of crowding - population density, measured as the number of people per square kilometre (based on the ZAMSTAR household enumeration). To better understand the relationships between community indicators and the ARI-to-prevalence ratio, we constructed corresponding graphical outputs (supplemental material) for ARI (S1) and prevalence separately (S2).

Ethics

Ethical approval for fieldwork was obtained through the CODA Study from University of Zambia Biomedical Research Ethics Committee (007-10-04), and LSHTM Ethics Committee (A211 3008).

Patient and Public Involvement

Community Advisory Boards (CABs) were consulted throughout the ZAMSTAR trial and formed before the implementation of interventions. In the sixteen Zambian communities, CABs were formed from pre-existing Neighbourhood Health Committees (NHCs) in each community. NHCs were already representative of different geographical 'zones' in the trial communities, endorsed by an Act of Parliament and democratically elected. NHCs were therefore asked if they would like to represent public (including patient) TB and HIV interests, and sought representation across interest groups, age and gender. Although not involved in the design of the ZAMSTAR trial, CABs were closely involved from ZAMSTAR baseline research onwards. Zambian research work was conducted by Zambart, a research organisation housed by the University of Zambia's School of Medicine and with links to the London School of Hygiene and Tropical Medicine. CAB members received training in Good Clinical Practice (GCP), research protocols and research dissemination. They were involved in the public randomisation of the trial communities and all research dissemination. Other than the CABs, representative participants involved in different components of the trial were also invited to community and district dissemination events. This included BBS disseminations, carried out at community level, using informative flyers for community members. Being a sub-study of ZAMSTAR, CODA used the same CAB structures and communication channels. The community engagement lead, Musonda Simwinga, used his experience with the ZAMSTAR CABs as a platform for his own PhD on

For peer review only

community engagement using CABs in trials, including reflections on equitable partnerships and accountability, and to develop other CAB structures for subsequent trials.[21]

RESULTS

Table 1 shows the relational categorisation of socio-spatial findings from the BBS CODA data i.e. it is a structured representation of the qualitative analysis. The qualitative findings are described below. Figure 1 merges socio-spatial variables with the calculation of transmission efficiency across study communities. It shows the relationship between the ARI-to-prevalence ratio (y axis) and twelve indicators (eleven qualitative and one quantitative). Similarly, Figures S1 and S2 separately show the same indicators in relation to ARI and TB prevalence, respectively. Indicators (variables) were typically more tightly-linked with ARI, and less tightly-linked with disease prevalence, than they were with the ARI-to-prevalence ratio. The stronger link between the indicators and ARI may reflect that ARI is a cumulative measure, based on relatively large numbers; whereas disease prevalence is a cross-sectional measure, with relatively small numbers of cases in each community that may fluctuate randomly over time.

Variations in Community Life

Certain communities were classified as more 'socially permeable' than others: socially 'open' to exchange across geographical boundaries and orientated towards mixing across age and gender in congregate settings. In nine communities, adults from other regions - 'outsiders' - engaged more with local community life. The presence of 'outsiders' appeared particularly diverse in three of these nine communities, where men, women, girls and boys visited. By contrast, there was little to no evidence of 'outsiders' operating in the other six communities. In places with a strong presence of 'outsiders', social mixing across age groups and genders appeared more evident, although where and how this contact took place varied. For example, in community 14, bars seemed to play more of a role in bringing people together than video shows. We also distinguished between communities where both adults and children travelled out and those where mostly adults travelled out. In four communities, only men travelled out regularly. This travel could be linked to livelihood pursuits, often of an agricultural nature. Involvement in seasonal farming or fishing was widespread across age and gender in four communities; in an additional seven communities, there was evidence of engagement with outdoor farming or fishing, but this was less widespread. Residents left communities for short periods to fish and/or buy fish or tend to fields in the rainy season. By contrast, the remaining four (of the fifteen) communities showed no evidence of involvement in farming or fishing.

For peer review only

Levels of crowding varied at health centres and in other gathering places, although the majority of communities were classified as generally 'crowded' by fieldworkers. The same was true for housing density: only five communities were observed to have relatively low density housing. Importantly, public transport was often overloaded and gauged as crowded, with extended durations of travel. Ventilation during travel was often poor given that it was common practise to keep windows shut to prevent dust. In one community, panel vans with no windows were being used. Although most communities were reliant on buses, minibuses and/or vans for travel, some were substantially less dependent on public transport; residents of one community mostly travelled by foot. When assessing who remained within the geographical boundaries of their residential community during the day, communities again differed. In six communities there was little evidence of residents remaining. Children and/or women generally remained in six of the other nine communities, but men rarely did. Only in three communities were local men more present within their community.

In all communities bar one, women spent a notable amount of time within the home. In certain communities, men appeared to be more engaged with recreational activity. Indoor recreational activities across communities were often similar, but contact patterns differed. Children came into contact with adults at drinking places and/or at pool table clubs in seven of the communities, while in the others this was far less evident. In three communities, only men were ever seen at bars. In certain communities, women were observed with babies strapped to their backs and boys were frequently trading in eggs and/or bottles, highlighting the multi-functionality of drinking places in these areas. Bars and taverns were generally crowded and poorly ventilated, with limited small windows designed to deter theft. Watching films in small makeshift tented 'theatres' was a popular leisure activity in fourteen communities. Shows were held in small outdoor structures, erected using poles and tarpaulin, measuring approximately 4 x 2.5m. These structures were darkened as much as possible to enhance viewing, by closing gaps in the tarpaulin, which in turn limited ventilation. Audiences were ordinarily seated on a few tightly packed benches. Children watched video shows during the day, and in the evening, adults and children often watched together. All communities showed widespread commitment to their local churches. Community members of all ages spent a substantial proportion of their time at services ranging between two and six hours, which often exceeded the time spent on many of the other contact activities identified. It should be noted that most community residents are Christian and the fieldwork was conducted in the period leading up to Easter.

Patterns with respect to transmission efficiency

For peer review only

Population density and qualitative assessments of crowding were broadly concordant, given the small sample size (number of communities). Communities sharing higher transmission efficiency could generally be classed as more crowded whereas communities with less transmission efficiency tended to present in more of a mixed way (across lower and higher degrees of crowding). Where transmission efficiency was highest, there were also high levels of social permeability. This is illustrated by four communities (3, 4, 7, 8) where a wide range of non-residents were present and resident adults frequently travelled out. Moreover, communities 4, 7 and 8 showed evidence of social mixing across age and gender in local recreational activity as well as having a number of residents who remained in the community during the day, suggesting a diverse range of contact opportunities. In terms of social mixing, Community 3 was distinctive. Whilst 'outsiders' came in and locals travelled out, residents, as a whole, were not remaining as much and social mixing during recreational activity appeared relatively limited. This reflects particular characteristics of the community. The community had a large local market, attracting outsiders, and a large proportion of established lower middle-class residents (including police and army) who travelled out to work, creating a substantial daily population 'swap'. Class distribution also undermined diversity in local social mixing (with middle-class having a preference for socialising outside of their community). Overall, the social permeability of these four higher efficiency communities contrasted with the lower transmission efficiency seen in the most socially closed communities, with far fewer 'outsiders' and less travel. Widespread participation in farming and/or fishing (across age and gender) seemed to correspond with only lower levels of transmission efficiency, while partial or no time spent farming/fishing corresponded with both high and low transmission efficiency. There was no pattern across indicators of wider participation in indoor activities, however, where only children watched video shows, transmission intensity appeared lower, with clear exceptions of communities 2 and 3.

DISCUSSION

This interdisciplinary analysis suggests that certain communities may have been protected from *Mtb* transmission in different and sociologically complex ways. Communities with lower measures of transmission efficiency included, but were not limited to: those in which fishing and/or farming were particularly dominant across gender and age; where public transport was more ventilated and less overloaded; where less crowding took place in public space; where fewer 'outsiders' were present; where only men mostly travelled out of their community; where there was negligible evidence of indoor recreational activity; and where population and housing density was lower. Links between some of these socio-spatial aspects and *Mtb* transmission risk have been shown, using different approaches, in other sub-Saharan settings. In Malawi, Odone et al. identified that TB was more

For peer review only

common amongst those working in the cash economy than those in subsistence economy[22], and studies in South Africa have highlighted the significance of confined public transport and gathering places, with emphasis on the implications for air hygiene and rebreathed air volumes.[23-25]

From a distinctly qualitative angle, our work begins to unveil how the sociological topography of a particular community could play an important role in shaping transmission efficiency. Two observations are significant in this regard. The first observation relates to the relative vulnerability of the communities with more outwardly orientated and socio-economically diverse features in comparison to their more socially secluded and/or agriculturally focused counterparts. Certain communities exhibit a dual dynamic of spatial crowding and 'social permeability', which could be providing increased opportunity for the importation of *Mtb* and local aerosol transmission in poorly ventilated spaces. 'Social permeability' not only reflects the presence of non-residents within the community, it also refers to the frequency with which local inhabitants travel out, providing additional opportunity for *Mtb* exposure and infection (or re-infection) outside the residential community. It seems that this dual dynamic of spatial 'closeness' and social 'openness' is relevant to increased *Mtb* transmission across the communities.

The second observation relates to a distinction that needs to be made between what we could call 'structural' and 'routine' variables. By structural variables we refer to socio-economic factors that are to a certain extent beyond people's agency. For instance, communities that are more structurally dependent on exchange and interaction with outsiders could be more vulnerable to *Mtb* exposure. Routine variables refer to habits or patterns of behaviour which although related to socio-economic factors are not obviously reducible to them. Watching video shows is a good example; it is a form of routine leisure activity which carries a particularly high presumptive risk for *Mtb* transmission because of the darkened, poorly ventilated and contained space in which it is conducted. For health policy, our distinction between 'structural' and 'routine' variables is important because the latter would be more malleable to intervention. For instance, whilst structural dependency on the outside world cannot be altered so easily, people can be encouraged to change the way in which they conduct routine leisure activities, or a concerted effort made to increase ventilation in these places.[26]

Our secondary analysis has limitations. Figure 1 is exploratory and hypothesis-generating and measures of association must be interpreted with appropriate caution. However, it does suggest tentative patterns with respect to some indicators worthy of attention in future work. This analysis does not report on all significant features linked to social and spatial engagement in the communities.

For peer review only

We do not pay much attention to the large amount of time residents spent in church as no substantial differences presented across communities, yet congregation of this nature has been established as a transmission risk factor in high burden settings.[27] Fieldwork was conducted at particular time of year and observations took place during the daytime, which may limit the generalisability of our observations – not only across but also within the study communities. We are also aware that local mines and prisons may have an important impact on *Mtb* transmission risk in some communities[28-32], however, we were unable to identify this in these data. Additionally, there was scant evidence of women or men regularly attending traditional ceremonies, which may require further consideration. It is possible that the observational BBS did not hold enough ethnographic depth to capture the pervasiveness of more privately guarded socio-spatial activities. Finally, the data set is not recent and we had to exclude one community due to poor data quality. These limitations are offset by the novel interdisciplinary endeavour, the unusual breadth of the data set and the continued relevance of socio-spatial context for *Mtb* transmission.

CONCLUSION

Localised sociological distinctions offered an explanation of why certain communities could have been more (or less) vulnerable to *Mtb* transmission. A dual dynamic of social permeability and spatial crowding appears relevant to increased transmission efficiency in specific Zambian communities. Interventions linked to these socio-spatial aspects might enhance community level resilience to TB, by reducing *Mtb* exposure and infection for local residents. A conceptual framework differentiating structural and routine elements of high-risk socio-spatial features would be a valuable foundation for the development of contextualised public health interventions. For example, some socio-spatial aspects carrying transmission risk are easily adjusted e.g. increasing ventilation on transport or at video shows. However, other more structural aspects are less malleable e.g. reliance on public transport.

ACKNOWLEDGEMENTS

We extend thanks to the communities, all participants, the regulatory authorities, the Ministry of Health and Zambart staff in Zambia for their support and involvement. In particular, Levy Chilikwela provided invaluable assistance with qualitative data analysis. We are also grateful to Patrick Baert, Peter Godfrey-Faussett and Jean Hunleth for feedback on various versions of this paper. This secondary analysis received no specific grant from any funding agency in the public, commercial or not-for-profit sectors. However: HA, VB, PJD, EJM and RGW were funded by the Bill and Melinda Gates Foundation (Consortium to Respond Effectively to the AIDS/TB Epidemic: 19790.01). RGW is funded

For peer review only - http://bmjopen.bmj.com/site/about/guidelines.xhtml

by the Wellcome Trust (218261/Z/19/Z), NIH (1R01AI147321-01), EDTCP (RIA208D-2505B), UK MRC (CCF17-7779 via SET Bloomsbury), ESRC (ES/P008011/1), BMGF (OPP1084276, OPP1135288 & INV-001754), and the WHO (2020/985800-0). PJD is supported by the UK Medical Research Council (MR/P022081/1). (This UK funded award is part of the EDCTP2 programme supported by the European Union.) The funders had no involvement in the design, collection, analysis or interpretation of the data, in writing the manuscript or in the decision to submit. No conflicts of interest declared.

Author Contributions

Substantial contribution to conception and design of ZAMSTAR: HA, VB, KS. Substantial contribution to conception and design of CODA: HA, VB, PJD, RW**.** Substantial contributions to conception and design of secondary analysis: VB, PJD, BM, EJM, RW. Acquisition of study data: HA, VB, PJD, KS. Analysis and interpretation of data: PJD, EJM. Drafting of article: EJM. Revising critically for intellectual content: HA, VB, PJD, BM, RW.

REFERENCES

1. Global tuberculosis report 2020. Geneva: World Health Organisation; 2020. Licence: CC BY-NC-SA 3.0 IGO. https://www.who.int/tb/publications/global_report. Accessed 22 October 2020.
2. Ayles H, Schaap A, Nota A, et al. Prevalence of Tuberculosis, HIV and Respiratory Symptoms in Two Zambian Communities: Implications for Tuberculosis Control in the Era of HIV. *PLoS One* 2009;4(5):e5602. DOI:10.1371/journal.pone.0005602.
3. Kapata N, Chanda-Kapata P, O'Grady J, et.al. Trends of Zambia's tuberculosis burden over the past two decades. *Tropical Medicine and International Health* 2011;16(11):1404-1409. https://doi.org/10.1111/j.1365-3156.2011.02849.x. Accessed 22 October 2020
4. Chanda-Kapata P, Kapata N, Klinkenberg E, et al. The prevalence of HIV among adults with pulmonary TB at a population level in Zambia. *BMC Infect Dis* 2017;17(236). DOI: 10.1186/s12879-017-2345-5.
5. Coffman J, Chanda-Kapata P, Marais BJ, et al. Tuberculosis among older adults in Zambia: burden and characteristics among a neglected group. *BMC Public Health* 2017;17(804). DOI:10.1186/s12889-017-4836-0.
6. Cremers AL, Manon de Laat M, Kapata N, et al. Assessing the Consequences of Stigma for Tuberculosis Patients in Urban Zambia. *PLoS One* 2015;10(3):e0119861. DOI:10.1371/journal.pone.0119861.
7. Bond V, Nyblade L. The importance of addressing the unfolding TB-HIV stigma in high HIV prevalence settings. *J Community Appl Soc Psychol* 2006;16:452-461. DOI:10.1002/casp.893.

8. Chanda D, Gosnell DJ. The Impact of Tuberculosis on Zambia and the Zambian Nursing Workforce. *Online J Issues Nurs* 2006;11(1):4. DOI: 10.3912/OJIN.Vol11No01Man03.
9. Murray EJ, Bond VA, Marais BJ, et al. High levels of vulnerability and anticipated stigma reduce the impetus for tuberculosis diagnosis in Cape Town, South Africa. *Health Policy Plan* 2013;28(4):410-418. DOI:10.1093/heapol/czs072.
10. Uys P, Marais BJ, Johnstone-Robertson S, et al. Transmission Elasticity in Communities Hyperendemic for Tuberculosis. *Clin Infect Dis* 2011;52(12):1399-404. DOI:10.1093/cid/cir229.
11. Hermans S, Horsburgh CR Jr., Wood R. A Century of Tuberculosis Epidemiology in the Northern and Southern Hemisphere: The Differential Impact of Control Interventions. *PLoS ONE* 2015;10(8):e0135179. DOI:10.1371/journal.pone.0135179.
12. Ayles H, Muyoyeta M, Du Toit E, et al. Effect of household and community interventions on the burden of tuberculosis in southern Africa: the ZAMSTAR community-randomised trial. *Lancet* 2013;382(9899):1183-1194. DOI:10.1016/S0140-6736(13)61131-9.
13. Sismandis C, Moulton LH, Ayles H, et al. Restricted Randomisation of ZAMSTAR: a 2 X 2 factorial cluster randomised trial. *Clinical Trials* 2008;5:316-327. DOI:10.1177/1740774508094747.
14. Ayles H, Sismanidis C, Beyers N, et al. ZAMSTAR, The Zambia South Africa TB and HIV Reduction study: Design of a 2 × 2 factorial community randomized trial. *Trials* 2008;9(63). DOI: https://doi.org/10.1186/1745-6215-9-63.
15. Shanaube K, Sismanidis C, Ayles H, et al. Annual Risk of Tuberculous Infection Using Different Methods in Communities with a High Prevalence of TB and HIV in Zambia and South Africa. *PLoS ONE* 2009;4(11):e7749. DOI: https://doi.org/10.1371/journal.pone.0007749.
16. Dodd PJ, Looker C, Plumb ID, et al. Age and Sex Specific Social Contact Patterns and Incidence of *Mycobacterium tuberculosis* Infection. *Am J Epidemiol* 2016;183(2):156-166. DOI: https://doi.org/10.1093/aje/kwv160.
17. Bond V, Ngwenya F, Murray E, et al. Value and Limitations of Broad Brush Surveys Used in Community-Randomized Trials in Southern Africa. *Qual Health Res* 2019;29(5):700-718. DOI:10.1177/1049732318809940.
18. Bond V. Terrains and Tuberculosis: The Model Applied in Urgent Public Health Settings. In: Wallman S, ed. The Capability of Places: Methods for Modelling Community Response to Intrusion and Change. London, UK: Pluto Press, 2011: 80-110.

For peer review only

19. Murray EJ. The Social Terrain of Endemic Tuberculosis in and around Cape Town [MPhil dissertation]. Faculty of Health Sciences, Stellenbosch University, South Africa; 2010. Online at: http://hdl.handle.net/10019.1/5387 . Accessed 22 October 2020.
20. Van Leth F, Van der Werf MJ, Borgdorff MW. Prevalence of tuberculous infection and incidence of tuberculosis; a re-assessment of the Styblo rule. *Bull World Health Organ* 2008;86(1):20-6. DOI:10.2471/blt.06.037804.
21. Simwinga M. Exploring the role of community participation in research studies: the case of two Community Advisory Boards in Zambia [doctoral dissertation]. London School of Hygiene and Tropical Medicine, United Kingdom; 2015.
22. Odone A, Crampin AC, Mwinuka V, et al. Association between socioeconomic position and tuberculosis in a large population-based study in rural Malawi. *PLoS One* 2013;8(10):e77740. DOI:https://doi.org/10.1371/journal.pone.0077740.
23. Andrews JR, Morrow C, Wood R. Modeling the role of public transportation in sustaining tuberculosis transmission in South Africa. *Am J Epidemiol* 2013;177(6):556–561. DOI:https://doi.org/10.1093/aje/kws331.
24. Issarow C, Mulder N, Wood R. Environmental and social factors impacting on epidemic and endemic tuberculosis: a modelling analysis. *R.Soc.open sci.* 2018 Jan;5(1). DOI:http://doi.org/10.1098/rsos.170726.
25. Andrews JR, Morrow C, Walensky RP, et al. Integrating Social Contact and Environmental Data in Evaluating Tuberculosis Transmission in a South African Township. *J Infect Dis* 2014;210(4):597–603. DOI:https://doi.org/10.1093/infdis/jiu138.
26. Richardson ET, Morrow CD, Kalil DB, et al. Shared air: a renewed focus on ventilation for the prevention of tuberculosis transmission [published correction appears in PLoS One. 2014; 9(8): e106216. *PLoS One*. 2014 May;9(5):e96334. DOI:10.1371/journal.pone.0096334.
27. Mathema B, Andrews JR, Cohen T, et al. Drivers of Tuberculosis Transmission. *J Infect Dis* 2017;216(suppl_6)S644-S653. DOI:10.1093/infdis/jix354.
28. Henostroza G, Topp SM, Hatwiinda S, et al. The High Burden of Tuberculosis (TB) and Human Immunodeficiency Virus (HIV) in a Large Zambian Prison: A Public Health Alert. *PLoS One* 2013 8(8): e67338. DOI: https://doi.org/10.1371/journal.pone.0067338.
29. O'Grady J, Hoelscher M, Atun R, et al. Tuberculosis in prisons in sub-Saharan Africa – the need for improved health services, surveillance and control. *Tuberculosis* 2011;91 (2):173-178.DOI: https://doi.org/10.1016/j.tube.2010.12.002.

For peer review only

30. Chanda-Kapata P, Osei-Afriyie D, Mwansa C, et al. Tuberculosis in the mines of Zambia: A case for intervention. *Asian Pacific Journal of Tropical Biomedicine* 2016; 6(9):803-807. DOI:https://doi.org/10.1016/j.apjtb.2016.06.015.

31. Stuckler D, Basu S, McKee M, et al. Mining and Risk of Tuberculosis in Sub-Saharan Africa. *Amer J Public Health* 2011;101: 524-530. DOI https://doi.org/10.2105/AJPH.2009.175646.

32. Godfrey-Faussett P, Sonnenberg P, Shearer SC et al. Tuberculosis control and molecular epidemiology in a South African gold-mining community. *Lancet* 2000; 356(9235):1066-1071. DOI: https://doi.org/10.1016/S0140-6736(00)02730-6

For peer review only

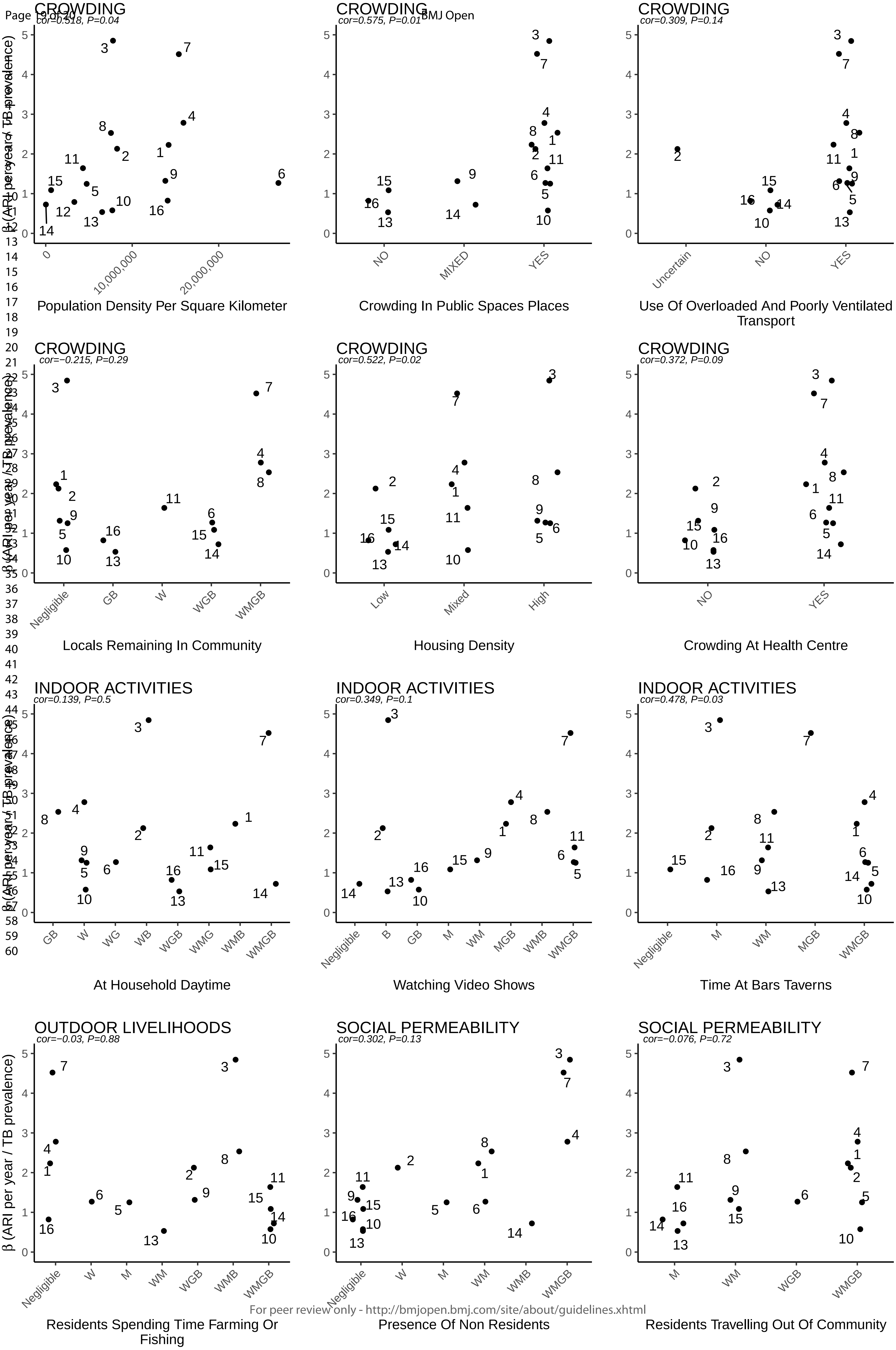
CROWDING
cor=0.518, P=0.04
β (ARI per year / TB prevalence)
Population Density Per Square Kilometer
0
10,000,000
20,000,000
CROWDING
cor=0.575, P=0.01
Crowding In Public Spaces Places
NO
MIXED
YES
CROWDING
cor=0.309, P=0.14
Use Of Overloaded And Poorly Ventilated Transport
Uncertain
NO
YES
CROWDING
cor=−0.215, P=0.29
Locals Remaining In Community
Negligible
GB
W
WGB
WMGB
CROWDING
cor=0.522, P=0.02
Housing Density
Low
Mixed
High
CROWDING
cor=0.372, P=0.09
Crowding At Health Centre
NO
YES
INDOOR ACTIVITIES
cor=0.139, P=0.5
At Household Daytime
GB
W
WG
WB
WGB
WMG
WMB
WMGB
INDOOR ACTIVITIES
cor=0.349, P=0.1
Watching Video Shows
Negligible
B
GB
M
WM
MGB
WMB
WMGB
INDOOR ACTIVITIES
cor=0.478, P=0.03
Time At Bars Taverns
Negligible
M
WM
MGB
WMGB
OUTDOOR LIVELIHOODS
cor=−0.03, P=0.88
Residents Spending Time Farming Or Fishing
Negligible
W
M
WM
WGB
WMB
WMGB
SOCIAL PERMEABILITY
cor=0.302, P=0.13
Presence Of Non Residents
Negligible
W
M
WM
WMB
WMGB
SOCIAL PERMEABILITY
cor=−0.076, P=0.72
Residents Travelling Out Of Community
M
WM
WGB
WMGB

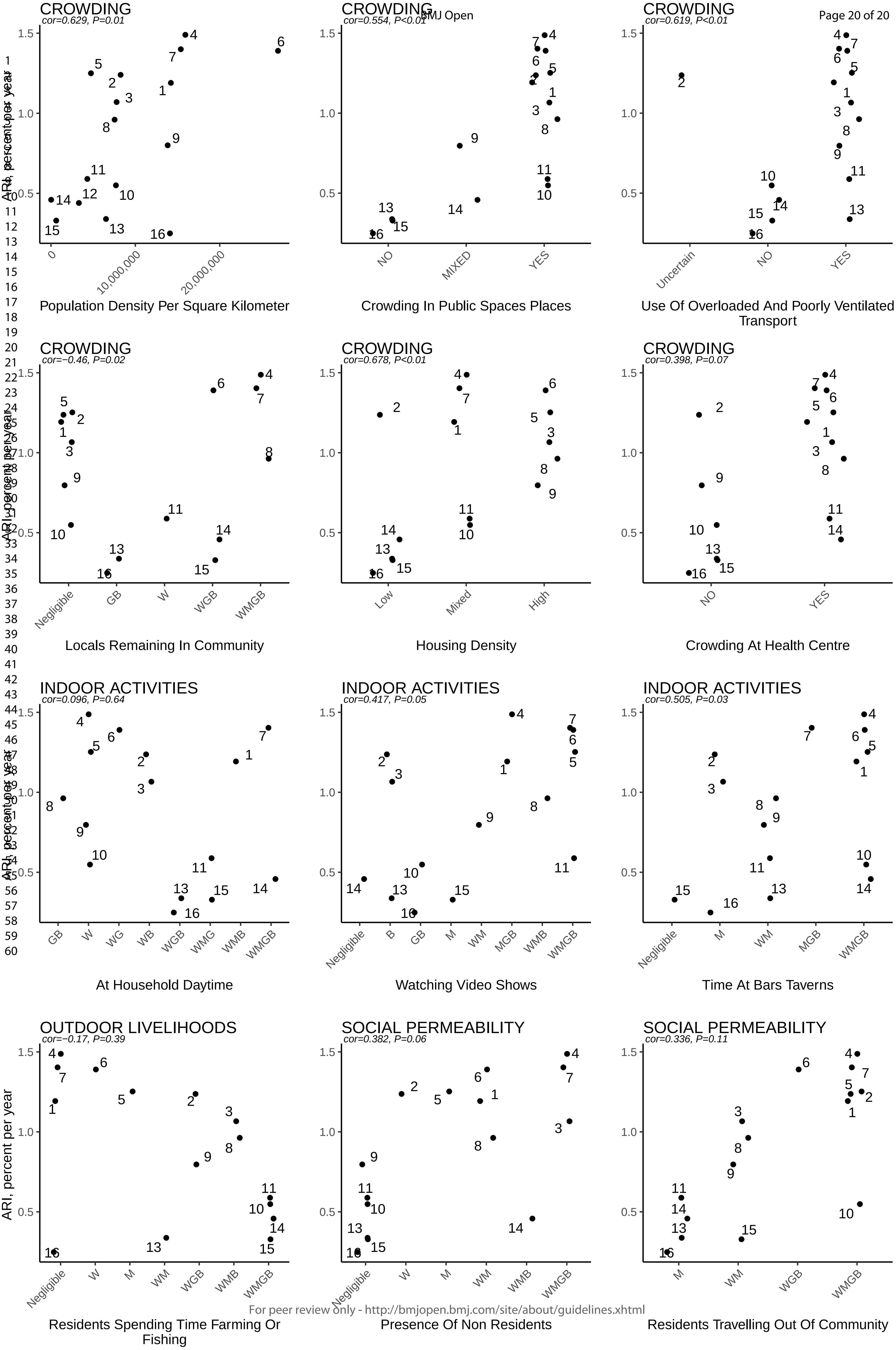
CROWDING
cor=0.629, P=0.01
ARI, percent per year
Population Density Per Square Kilometer
0
10,000,000
20,000,000
CROWDING
cor=0.554, P<0.01
Crowding In Public Spaces Places
NO
MIXED
YES
CROWDING
cor=0.619, P<0.01
Use Of Overloaded And Poorly Ventilated Transport
Uncertain
NO
YES
CROWDING
cor=−0.46, P=0.02
Locals Remaining In Community
Negligible
GB
W
WGB
WMGB
CROWDING
cor=0.678, P<0.01
Housing Density
Low
Mixed
High
CROWDING
cor=0.398, P=0.07
Crowding At Health Centre
NO
YES
INDOOR ACTIVITIES
cor=0.096, P=0.64
At Household Daytime
GB
W
WG
WB
WGB
WMG
WMB
WMGB
INDOOR ACTIVITIES
cor=0.417, P=0.05
Watching Video Shows
Negligible
B
GB
M
WM
MGB
WMB
WMGB
INDOOR ACTIVITIES
cor=0.505, P=0.03
Time At Bars Taverns
Negligible
M
WM
MGB
WMGB
OUTDOOR LIVELIHOODS
cor=−0.17, P=0.39
Residents Spending Time Farming Or Fishing
Negligible
W
M
WM
WGB
WMB
WMGB
SOCIAL PERMEABILITY
cor=0.382, P=0.06
Presence Of Non Residents
Negligible
W
M
WM
WMB
WMGB
SOCIAL PERMEABILITY
cor=0.336, P=0.11
Residents Travelling Out Of Community
M
WM
WGB
WMGB

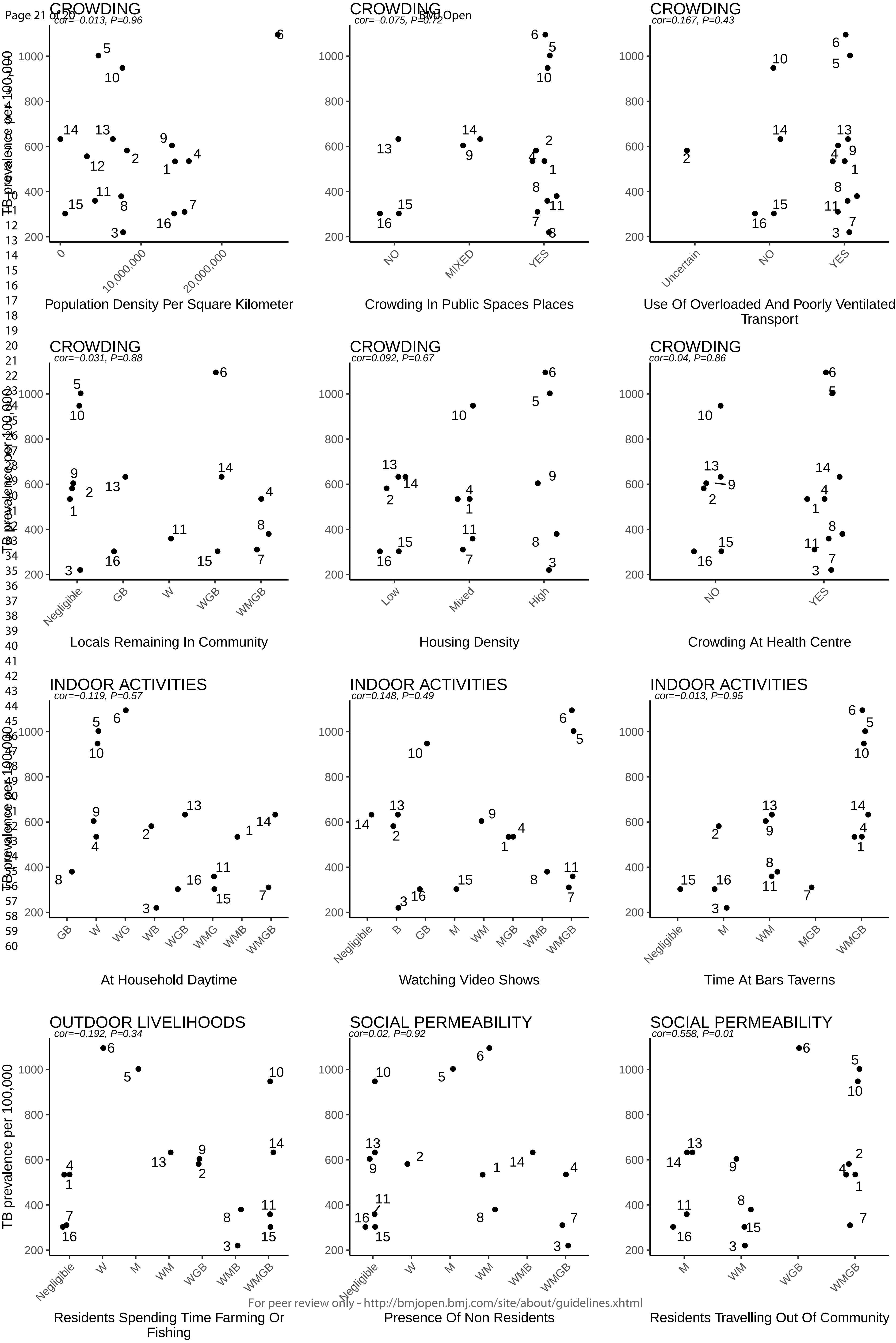
CROWDING
cor=−0.013, P=0.96
Population Density Per Square Kilometer
CROWDING
cor=−0.075, P=0.72
Crowding In Public Spaces Places
CROWDING
cor=0.167, P=0.43
Use Of Overloaded And Poorly Ventilated Transport
CROWDING
cor=−0.031, P=0.88
Locals Remaining In Community
CROWDING
cor=0.092, P=0.67
Housing Density
CROWDING
cor=0.04, P=0.86
Crowding At Health Centre
INDOOR ACTIVITIES
cor=−0.119, P=0.57
At Household Daytime
INDOOR ACTIVITIES
cor=0.148, P=0.49
Watching Video Shows
INDOOR ACTIVITIES
cor=−0.013, P=0.95
Time At Bars Taverns
OUTDOOR LIVELIHOODS
cor=−0.192, P=0.34
Residents Spending Time Farming Or Fishing
SOCIAL PERMEABILITY
cor=0.02, P=0.92
Presence Of Non Residents
SOCIAL PERMEABILITY
cor=0.558, P=0.01
Residents Travelling Out Of Community
TB prevalence per 100,000

BMJ Open

Sociological Variety and the Transmission Efficiency of Mycobacterium Tuberculosis: A Secondary Analysis of Qualitative and Quantitative Data from Fifteen Communities in Zambia.

Journal:	*BMJ Open*
Manuscript ID	bmjopen-2020-047136.R1
Article Type:	Original research
Date Submitted by the Author:	05-Jul-2021
Complete List of Authors:	Murray, Emma; Previously affiliated with The Desmond Tutu TB Centre, Stellenbosch Univeristy<br>Dodd, PJ; The University of Sheffield, School of Health and Related Research<br>Marais, B; The University of Sydney Children's Hospital Westmead Clinical School<br>Ayles, Helen; London School of Hygiene & Tropical Medicine, Clinical Research Department, Faculty of Infectious and Tropical Diseases; University of Zambia, Zambart, School of Public Health, University of Zambia<br>Shanaube, Kwame; University of Zambia, Zambart, School of Public Health, University of Zambia<br>Schaap, Albertus; London School of Hygiene & Tropical Medicine, Department of Infectious Disease Epidemiology<br>White, RG; London School of Hygiene & Tropical Medicine, TB Modelling Group, Department of Infectious Disease Epidemiology<br>Bond, Virginia; London School of Hygiene & Tropical Medicine, Department of Global Health and Development, Faculty of Public Health and Policy; University of Zambia, Zambart, School of Public Health
**Primary Subject Heading**:	Infectious diseases
Secondary Subject Heading:	Qualitative research, Epidemiology, Global health, Public health
Keywords:	Tuberculosis < INFECTIOUS DISEASES, SOCIAL MEDICINE, QUALITATIVE RESEARCH, PUBLIC HEALTH, Tropical medicine < INFECTIOUS DISEASES

SCHOLARONE™
Manuscripts

I, the Submitting Author has the right to grant and does grant on behalf of all authors of the Work (as defined in the below author licence), an exclusive licence and/or a non-exclusive licence for contributions from authors who are: i) UK Crown employees; ii) where BMJ has agreed a CC-BY licence shall apply, and/or iii) in accordance with the terms applicable for US Federal Government officers or employees acting as part of their official duties; on a worldwide, perpetual, irrevocable, royalty-free basis to BMJ Publishing Group Ltd ("BMJ") its licensees and where the relevant Journal is co-owned by BMJ to the co-owners of the Journal, to publish the Work in this journal and any other BMJ products and to exploit all rights, as set out in our licence.

The Submitting Author accepts and understands that any supply made under these terms is made by BMJ to the Submitting Author unless you are acting as an employee on behalf of your employer or a postgraduate student of an affiliated institution which is paying any applicable article publishing charge ("APC") for Open Access articles. Where the Submitting Author wishes to make the Work available on an Open Access basis (and intends to pay the relevant APC), the terms of reuse of such Open Access shall be governed by a Creative Commons licence – details of these licences and which Creative Commons licence will apply to this Work are set out in our licence referred to above.

Other than as permitted in any relevant BMJ Author's Self Archiving Policies, I confirm this Work has not been accepted for publication elsewhere, is not being considered for publication elsewhere and does not duplicate material already published. I confirm all authors consent to publication of this Work and authorise the granting of this licence.

For peer review only

Sociological Variety and the Transmission Efficiency of Mycobacterium Tuberculosis: A Secondary Analysis of Qualitative and Quantitative Data from Fifteen Communities in Zambia.

Emma J Murray[1], Peter J Dodd[2], Ben Marais[3], Helen Ayles[4,5], Kwame Shanaube[4], Albertus Schaap[6], Richard G White[7], Virginia Bond[4,8]

1. Desmond Tutu TB Centre, Stellenbosch University, Tygerberg, Cape Town, South Africa (previously).
2. School of Health and Related Research, University of Sheffield, Sheffield, United Kingdom.
3. Clinical School, Children's Hospital at Westmead, University of Sydney, Sydney, Australia.
4. Zambart, School of Public Health, University of Zambia, Lusaka, Zambia.
5. Clinical Research Department, Faculty of Infectious and Tropical Diseases, London School of Hygiene and Tropical Medicine, United Kingdom
6. Department of Infectious Disease Epidemiology, London School of Hygiene and Tropical Medicine, United Kingdom.
7. TB Modelling Group, Department of Infectious Disease Epidemiology, London School of Hygiene and Tropical Medicine, United Kingdom.
8. Department of Global Health and Development, Faculty of Public Health and Policy, London School of Hygiene and Tropical Medicine, United Kingdom.

For peer review only

Abstract: 300 words
Main Text: (excluding title page, abstract, summary, acknowledgements, references, figures and table): 4352 words
Number of References: 37
Table 1: Relational Categorisation of Socio-Spatial Tendencies across Communities
Figure 1: Transmission Efficiency (ratio of Annual Risk of Infection to Disease Prevalence) by Socio-Spatial Variables
Supplemental Material:
Annual Risk of Infection by Socio-Spatial Variables (S1)
Prevalence of Disease by Socio-Spatial Variables (S2)

Corresponding Author:
Emma Jane Murray
83 Highsett
Cambridge
CB2 1NZ
United Kingdom
emmajane.murray@gmail.com

ABSTRACT

Objectives: Selected Zambian communities formed part of a cluster randomised trial; the Zambia and South Africa TB and AIDS Reduction (ZAMSTAR) study. There was wide variability in the prevalence of *Mycobacterium Tuberculosis* (*Mtb*) infection and tuberculosis (TB) disease across these communities. We sought to clarify whether specific communities could have been more/less vulnerable to *Mtb* transmission as a result of sociological variety relevant to transmission efficiency.

Design: We conducted a mixed methods secondary analysis using existing sets of data. Firstly, we analysed qualitative data to categorise and synthesise patterns of socio-spatial engagement across communities. Secondly, we compared emergent sociological variables with a measure of transmission efficiency: the ratio of the annual risk of infection to TB prevalence.

Setting: ZAMSTAR communities in urban and peri-urban Zambia, spanning five provinces.

Participants: Fifteen communities, each served by a health facility offering tuberculosis treatment to a population of at least 25,000. TB notification rates were at least 400 per 100,000 per annum and estimates of HIV seroprevalence high.

Results: Crowding, movement, livelihoods and participation in recreational activity differed across communities. Based on twelve socio-spatial indicators, communities were qualitatively classified as more/less spatially crowded and as more/less socially 'open' to contact with others, with implications for the presumptive risk of *Mtb* transmission. For example, watching video shows in poorly ventilated structures posed a presumptive risk in more socially open communities, while outdoor farming and/or fishing were particularly widespread in communities with lower transmission measures.

Conclusions: A dual dynamic of 'social permeability' and crowding appeared relevant to disparities in *Mtb* transmission efficiency. To reduce transmission, certain socio-spatial aspects could be adjusted (e.g. increasing ventilation on transport), whilst more structural aspects are less malleable (e.g. reliance on public transport). We recommend integrating community level typologies with genome sequencing techniques to further explore the significance of 'social permeability'.

Key Words: tuberculosis transmission, community context, interdisciplinary study

For peer review only

SUMMARY

Strengths and Limitations of this study:

- We employed a novel qualitative approach to investigate disparities in transmission efficiency.
- We creatively combined population (community) level socio-spatial data with actual measurement of TB infection and disease.
- Data were effectively cross-sectional, focusing on interactions within localised geographical boundaries, and did not capture details of extra-community travel, nor of infrequent and/or seasonal events, potentially relevant to TB transmission.
- Results are context specific, therefore further investigations in other settings would be valuable to assess the generalisability of key concepts.
- Measures of association should be interpreted with appropriate caution and the study regarded as hypothesis generating.

INTRODUCTION

Interactions between the tuberculosis (TB) and human immunodeficiency virus (HIV) epidemics have complicated and compromised TB containment efforts in Zambia and other high burden countries. There is evidence that as the HIV epidemic ages, an increasing burden of undiagnosed TB is emerging amongst older adults in Zambia, underscoring the resilience of epidemic TB in the region. [1 – 5] Understanding the role of socio-economic factors in facilitating the transmission of *Mycobacterium Tuberculosis* (*Mtb)*, within and outside households, is critical to developing alternative approaches that could reduce ongoing *Mtb* transmission at the community level. [6-11] This inquiry explores how socio-spatial aspects of every-day community life might be relevant to differing *Mtb* transmission dynamics. We used a novel mixed methods interdisciplinary approach to conduct a secondary analysis of Zambian data sets, collected during a cluster randomised trial (CRT) - the Zambia and South Africa TB and AIDS Reduction Study (ZAMSTAR) - and as part of an ancillary study, named the Contact Observations of Daily Activities (CODA). [12, 13] The approach combined community level qualitative data, focused on the use of space and daily social interactions, with quantitative measurements of TB prevalence and annual risk of infection (ARI), to investigate whether differences in socio-spatial aspects across communities corresponded to a higher or lower *Mtb* transmission efficiency (measured as the ARI per prevalent TB case in a community).

ZAMSTAR (2004-2011) compared two interventions to reduce *Mtb* transmission and TB prevalence across Zambia and South Africa at a population level. The two interventions - enhanced community

For peer review only

TB case-finding and household level combined TB-HIV care - were implemented from 2006 to 2009. In Zambia, sixteen communities were selected by TB notification rates in excess of 400/100,000 per annum and a high HIV prevalence. [12, 14, 15, 16] Baseline prevalence of *Mtb* infection, as measured by tuberculin skin test (TST) surveys in primary school children, was highly variable, ranging from two to thirteen percent across the Zambian communities and allowed for the estimation of ARI. [14] The final ZAMSTAR prevalence survey found large variation in culture-confirmed TB prevalence, from 221 to 1095 per 100,000 in the Zambian communities. This variability implied, in part, differences in *Mtb* transmission dynamics. Towards the end of ZAMSTAR, CODA sought to measure patterns of social contacts relevant to *Mtb* transmission and model the implied ARIs in adult age groups. [13] Our inquiry supplements the CODA quantitative analysis of adult social contacts, investigating social and spatial contextual variables that may influence community level *Mtb* transmission.

METHODS

This secondary analysis used whole communities, rather than the individual, as a unit for analysis and drew on four sources of data: qualitative data collected for the purpose of the CODA study; estimates of population density from the ZAMSTAR household enumeration; estimates of ARI from ZAMSTAR TST surveys; and TB prevalence data from final ZAMSTAR TB prevalence survey. Details of ZAMSTAR household enumeration, TST data and implied ARI estimates, TB prevalence data and linked methods have been published. [12, 14, 16] The Zambian communities we investigated in this secondary analysis were pre-selected as a set of sixteen for the ZAMSTAR trial. The ZAMSTAR research design needed the potential to detect statistically significant reductions in TB prevalence and infection incidence. Therefore, communities were selected based on pre-specified criteria including TB case notification > 400/100,000. A further prerequisite was that TB diagnostic and treatment service were broadly equivalent (*de facto* functioning local health facilities provided by the state, with trained health care providers and voluntary TB supporters, serving a population of at least 25,000). Given the role of HIV infection in driving TB cases in this setting, ZAMSTAR additionally selected on the basis of relatively high burdens of HIV (compared to the national average). In the absence of population level data on HIV, proxy information was used to identify communities, drawing on the opinion of local experts, other survey sources and seeking the buy-in and advice from the Ministry of Health. The resulting Zambian ZAMSTAR communities were distributed across five provinces and districts with subsequent measures by the ZAMSTAR trial showing that HIV prevalence ranged between 8.1 and 26.6%. [12] All communities were predominantly high density urban communities (three were peri-urban) located along the line of rail and/or major trading routes. All communities had a broad mix of ethnic groups and four communities had a stronger, yet insignificant presence of non-nationals due to the proximity

For peer review only - http://bmjopen.bmj.com/site/about/guidelines.xhtml

of international borders and location within the capital city. Socio-economic status was mainly classified as low, with some pockets of lower middle-class in all communities.

We focus on the CODA qualitative data collection and analysis used for the identification of socio-spatial variables; our use of quantitative ARI and prevalence data from ZAMSTAR; and the mixed method analysis approach that investigated relationships between socio-spatial variables and calculated *Mtb* 'transmission efficiency'.

Qualitative Data

Following the intervention period of ZAMSTAR, Broad Brush Surveys (BBS) were conducted in 2011 across Zambian ZAMSTAR communities as part of CODA. [17] BBS is a method that lends itself to the systematic sociological comparison of bounded urban places (in this instance communities) across socio-space. The development of meta-indicators around a core research question allows for abstraction and a degree of generalisability. [17 -20] The CODA BBS aimed to a) update previous BBS data collected as part of ZAMSTAR between 2004 and 2005 [19] and b) provide insight into patterns of behaviour and settings of adult-to-child social mixing, in order to inform the design of a quantitative questionnaire.[13] The BBS fieldwork was completed by thirty-two research assistants trained over a two-day period in participatory methods. Fieldwork was conducted by a team of two (usually one man and one woman) over a period of five days in each community, using local languages. In sequence, each pair carried out a transect walk through the hub of the community; structured observations of different gender and age spaces (older men/women, younger men/women, boys/girls); a structured observation of the health facility, church services and the main transport depot; and day long household observations (two per community). The focus of these observations were presence and contact between adults and children. Daily activity charts were drawn up with boys and girls during the observations, in their spaces. Each team documented observations and conversations in the form of a written 'activity report'. Activity reports were semi-structured, open-ended and written in English, with translation completed in the process of writing by the relevant fieldworkers. Research teams were debriefed on a daily basis, both within the pair of research assistants and remotely over the phone. The second and last authors were part of the training and fieldwork.

The qualitative BBS data indicated how public places and transport were used by different age groups and genders within each community. Our focus on the broader community is justified as we know that transmission is less likely to take place within households in this particular setting. [21] For the purposes of this analysis, we identified and categorised patterns of social and spatial engagement using conversational and observational material from the BBS activity reports, until a point of

For peer review only - http://bmjopen.bmj.com/site/about/guidelines.xhtml

saturation was reached for each community. This was done in frequent consultation with a key member of the fieldwork team, who also guided the first author in conducting a relational comparison of the patterns identified across communities. One of the sixteen Zambian ZAMSTAR communities was dropped from our analysis due to poor data quality. Similarities and differences were subsequently compared across the remaining fifteen communities. We configured findings into an annotated tabular format of socio-spatial tendencies, broken down across age and gender categories where appropriate (Table 1).

For peer review only

TABLE 1: Relational Categorisation of Socio-Spatial Tendencies across Communities

Community ID Number	1	2	3	4	5	6	7	8	9	10	11	13	14	15	16
INDICATORS OF CROWDING:															
General Crowding in Public Spaces/ Places	YES	YES	YES	YES	YES	YES	YES	YES	Mixed	YES	YES	NO	Mixed	NO	NO
Use of Overloaded and Poorly Ventilated Transport	YES	Uncertain	YES	YES	YES	YES	YES	YES	YES	NO	YES	YES	NO	NO	NO
Locals Remaining in Community (daytime)	Negligible	Negligible	Negligible	M W B G	Negligible	W G B	M W G B	M W B G	Negligible	Negligible	W	B G	W G B	W G B	B G
Housing Density	Mixed	Low	High	Mixed	High	High	Mixed	High	High	Mixed	Mixed	Low	Low	Low	Low
Crowding at Health Centre	YES	NO	YES	YES	YES	YES	YES	YES	NO	NO	YES	NO	YES	NO	NO
Crowding at Churches	YES	YES	NO	YES	YES	YES	YES	YES	YES	YES	NO	YES	NO	NO	NO
PARTICIPATION IN INDOOR ACTIVITY:															
At Household (daytime)	M W B	W B	W B	W	W	W G	M W B G	B G	W	W	M W G	W B G	M W B G	M W G	W B G
Watching Video Shows	M G B	B	B	M G B	M W B G	M W B G	M W B G	M W B	WM	GB	M W B G	B	Negligible	M	GB
Time at Bars/Taverns	M W B G	M	M	M W B G	M W B G	M W B G	M G B	M W	M W	M W B G	M W	M W	M W B G	Negligible	M
To Church	M W B G	M W B G	M W B G	M W B G	M W B G	M W B G	M W B G	M W B G	M W B G	M W B G	M W B G	M W B G	M W B G	M W B G	M W B G
INDICATORS OF ECONOMY:															
Residents Farming / Fishing	Negligible	W G B	W M B	Negligible	M	W	Negligible	W M B	W G B	M W B G	M W B G	W M	M W B G	M W B G	Negligible
Residents at local market	M W	M	Negligible	M G B	W B	M G B	W G B	M W	W	W	W G	W	Negligible	M W	W
Residents using Local Shops/ Stalls	M	Negligible	M W	M G B	M	M W	M	M	Negligible	M	Negligible	M	M	M	M
INDICATORS OF SOCIAL PERMEABILITY:															
Presence of Non-Residents	M W	W	M W B G	M W B G	M	M W	M W B G	M W	Negligible	Negligible	Negligible	Negligible	W M B	Negligible	Negligible
Residents Travelling Out of Community	M W B G	M W B G	M W	M W B G	M W B G	M W B G	M W B G	M W	M W	M W B G	M	M	M	M W	M
Residents Travelling to External Market	M W	Negligible	W	M W	W	M W	M W	Negligible	W	W	Negligible	W	Negligible	W	W
Social Mixing (general) across Age and Gender	Some	YES	YES	YES	YES	Some	Some	YES	Some	YES	Least	Some	YES	Less	Least
At Health Centre	M W B G	Negligible	M W B G	W G B	M W B G	M W B G	W G B	M W B G	W	W	M W B G	W G B	W G B	W	W
OTHER:															
Children Not Attending School	Negligible	Negligible	Negligible	B G	B G	Negligible	B G	Negligible	Negligible	Negligible	Negligible	Negligible	Negligible	Negligible	B G
To Salon/Barber	W	Negligible	Negligible	W	Negligible	M W G	W	Negligible	W	W	W	Negligible	W	W	W
To Outdoor Water Point	W G	Negligible	Negligible	W G	W	Negligible	W G B	W	Negligible	W G	Negligible	G	Negligible	Negligible	W
To Outdoor Recreation	B	B	M	M B G	M B	Negligible	M B G	B	Negligible	B	B G	B	M B	M B	B G

LEGEND:
Rows of variables were identified and thematically grouped following qualitative BBS data analysis. Themes are capitalised.
Each column provides a ‘snapshot’ of general tendencies within a particular community. Qualitative assessments of variables were relationally gauged across communities, using BBS data.
M = Men
W = Women
B = Boys
G = Girls

For peer review only - http://bmjopen.bmj.com/site/about/guidelines.xhtml

For peer review only

We delineated the strongest variables as broadly linked to one or more of the following aspects: 1) levels of spatial crowding, pertaining to the amount of people within shared/confined spaces and the density of physical structures; 2) the range and degree of participation in predominantly indoor recreational activity; 3) the dominance of outdoor livelihoods e.g. widespread commitment to farming or fishing dramatically shaped socio-spatial engagement in certain communities; and 4) what we have termed as 'social permeability' i.e. the level of exchange between, or movement of, local residents and non-residents across and within geographical boundaries, with some communities clearly more socially 'open' to extra-community contact. Eleven qualitative indicators were identified to represent assessments of socio-spatial variability, selected on the basis of being observable (tangible) and categorical, with perceived relevance for TB transmission. The indicators were: general crowding in public spaces/places; the use of overloaded and poorly ventilated transport; local residents remaining in their residential community during the day; housing density; crowding at health centres; time spent at home during the day; watching video shows; going to bars/taverns; spending time farming/ fishing; the presence of non-residents; and evidence of residents travelling out of their residential community.

Quantitative Analysis

As part of ZAMSTAR, TST surveys were conducted in a cohort of primary school children to calculate ARI across communities, estimating community ARIs of between 0.25%/year and 1.49%/year (using a 15mm cut off for positivity). [16] For our mixed method analysis, we calculated the ARI-to-TB prevalence ratio, which is an established measure of 'transmission efficiency' (beta, in infectious disease modelling) and likely to be influenced by social features that affect the patterns and intensity of mixing relevant to *Mtb* transmission. Karel Styblo used this ratio to help describe historical patterns in European TB epidemiology. While the values that Styblo found no longer apply, the ratio remains a useful metric.[22] We calculated this ratio for each community using calculated ARI point estimates and TB prevalence point estimates determined during ZAMSTAR. We also computed Kendall Rank correlation coefficients (Spearman correlation coefficient for the quantitative indicator, population density) and approximate P-values. Given the number of variables considered, it is not appropriate to consider P-values below a certain threshold as 'significant'; they simply provide some guidance on how likely it is an apparent individual pattern could arise by chance.

Mixed Methods Analysis

After completing the qualitative analysis and quantitative calculations described above, we first correlated the measure of transmission efficiency with the eleven qualitative variables. Each variable was titled according to one of the four social aspects to which it most strongly indicated. For example,

For peer review only

the variable 'Presence of Non-Residents' was regarded as a strong indicator of social permeability, given the manner in which it presented in the BBS. Qualitative variables (indicators) were also ranked by a priori expectations of increasing transmission risk. For example, "high" > "low" for housing density, or "WMGB" > "M" for women, men, girls and boys spending time in bars versus only men. Previous work on these communities combining the TB prevalence results, ARI estimates and quantitative social contact data, suggested men are responsible for more infections than women. [13] Children, especially age <5 years, typically have lower bacteriological load than adults and are likely to contribute less to transmission than adults of either sex.". We also included an objective quantitative indicator of crowding - population density, measured as the number of people per square kilometre (based on the ZAMSTAR household enumeration). To better understand the relationships between community indicators and the ARI-to-prevalence ratio, we constructed corresponding graphical outputs (supplemental material) for ARI (S1) and prevalence separately (S2).

Patient and Public Involvement

Community Advisory Boards (CABs) were consulted throughout the ZAMSTAR trial and formed before the implementation of interventions. In the sixteen Zambian communities, CABs were formed from pre-existing Neighbourhood Health Committees (NHCs) in each community. NHCs were already representative of different geographical 'zones' in the trial communities, endorsed by an Act of Parliament and democratically elected. NHCs were therefore asked if they would like to represent public (including patient) TB and HIV interests, and sought representation across interest groups, age and gender. Although not involved in the design of the ZAMSTAR trial, CABs were closely involved from ZAMSTAR baseline research onwards. Zambian research work was conducted by Zambart, a research organisation housed by the University of Zambia's School of Medicine and with links to the London School of Hygiene and Tropical Medicine. CAB members received training in Good Clinical Practice (GCP), research protocols and research dissemination. They were involved in the public randomisation of the trial communities and all research dissemination. Other than the CABs, representative participants involved in different components of the trial were also invited to community and district dissemination events. This included BBS disseminations, carried out at community level, using informative flyers for community members. Being a sub-study of ZAMSTAR, CODA used the same CAB structures and communication channels. The community engagement lead, Musonda Simwinga, used his experience with the ZAMSTAR CABs as a platform for his own PhD on community engagement using CABs in trials, including reflections on equitable partnerships and accountability, and to develop other CAB structures for subsequent trials. [23]

For peer review only

RESULTS

Table 1 shows the relational categorisation of socio-spatial findings from the BBS CODA data i.e. it is a structured representation of the qualitative analysis. The qualitative findings are described below. Figure 1 merges socio-spatial variables with the calculation of transmission efficiency across study communities. It shows the relationship between the ARI-to-prevalence ratio (y axis) and twelve indicators (eleven qualitative and one quantitative). Similarly, Figures S1 and S2 separately show the same indicators in relation to ARI and TB prevalence, respectively. Indicators (variables) were typically more tightly-linked with ARI, and less tightly-linked with disease prevalence, than they were with the ARI-to-prevalence ratio. The stronger link between the indicators and ARI may reflect that ARI is a cumulative measure, based on relatively large numbers; whereas disease prevalence is a cross-sectional measure, with relatively small numbers of cases in each community that may fluctuate randomly over time.

Variations in Community Life

Certain communities were classified as more 'socially permeable' than others: socially 'open' to exchange across geographical boundaries and orientated towards mixing across age and gender in congregate settings. In nine communities, adults from other regions - 'outsiders' - engaged more with local community life. The presence of 'outsiders' appeared particularly diverse in three of these nine communities, where men, women, girls and boys visited. In contrast, there was little to no evidence of non-residents operating in the other six communities. In places with a strong presence of 'outsiders', social mixing across age groups and genders appeared widespread, although where and how this contact took place varied. For example, in community 14, bars played a greater role in bringing people together than video shows. We also distinguished between communities where both adults and children travelled out and those where mostly adults travelled out. In four communities, only men travelled out regularly. This travel could be linked to livelihood pursuits, often of an agricultural nature. Involvement in seasonal farming or fishing was particularly widespread across age and gender in four communities; in an additional seven communities, there was evidence of engagement with outdoor farming or fishing, but this was notably less intense. Residents left communities for short periods to fish and/or buy fish or tend to fields in the rainy season. In contrast, the remaining four (of the fifteen) communities showed no evidence of involvement in farming or fishing.

Levels of crowding varied at health centres and in other gathering places, although the majority of communities were classified as generally 'crowded' by fieldworkers. The same was true for housing

For peer review only

density: only five communities were observed to have relatively low density housing. Importantly, public transport was often overloaded and gauged as crowded, with extended durations of travel. Ventilation during travel was often poor given that it was common practise to keep windows shut to prevent dust. In one community, panel vans with no windows were being used. Although most communities were reliant on buses, minibuses and/or vans for travel, some were substantially less dependent on public transport; residents of one community mostly travelled by foot. When assessing who remained within the geographical boundaries of their residential community during the day, communities again differed. In six communities there was little evidence of residents remaining. Children and/or women generally remained in six of the other nine communities, but men rarely did. In three particular communities local men were significantly more present.

In all communities bar one, women spent time within the home. In certain communities, men appeared to be more engaged with recreational activity. Indoor recreational activities across communities were often similar, but contact patterns differed. Children came into contact with adults at drinking places and/or at pool table clubs in seven of the communities, while in the others this was far less evident. In three communities, only men were ever seen at bars. In certain communities, women were observed with babies strapped to their backs and boys were frequently trading in eggs and/or bottles, highlighting the multi-functionality of drinking places in these areas. Bars and taverns were generally crowded and poorly ventilated, with limited small windows designed to deter theft. Watching films in small makeshift tented 'theatres' was a popular leisure activity in fourteen communities. Shows were held in small outdoor structures, erected using poles and tarpaulin, measuring approximately 4 x 2.5m. These structures were darkened as much as possible to enhance viewing, by closing gaps in the tarpaulin, which in turn limited ventilation. Audiences were ordinarily seated on a few tightly packed benches. Children watched video shows during the day, and in the evening, adults and children often watched together. All communities showed widespread commitment to their local churches. Community members of all ages spent a substantial proportion of their time at services ranging between two and six hours, which often exceeded the time spent on many of the other contact activities identified. It should be noted that most community residents are Christian and the fieldwork was conducted in the period leading up to Easter.

Patterns with respect to transmission efficiency

Population density and qualitative assessments of crowding were broadly concordant, given the small sample size (number of communities). Communities sharing higher transmission efficiency could generally be classified as more crowded whereas communities with lower transmission efficiency

For peer review only

tended to present in more of a mixed way (across lower and higher degrees of crowding). By tracing an individual community (number) through Figure 1, one is able to consider the interaction of the different socio-spatial variables identified. Where transmission efficiency was highest, there were also high levels of social permeability. This is illustrated by four communities (3, 4, 7, 8) where a wide range of non-residents were present and resident adults frequently travelled out. Moreover, communities 4, 7 and 8 showed evidence of social mixing across age and gender in local recreational activity as well as having a number of residents who remained in the community during the day, suggesting a diverse range of contact opportunities. In terms of social mixing, Community 3 was distinctive. Whilst 'outsiders' came in and locals travelled out, there was a significant pattern of extra-community travel and many locals did not remain in the community during the day. Furthermore, social mixing during recreational activity appeared relatively limited. This reflects particular characteristics of the community. The community had a large local market, attracting outsiders, and a large proportion of established lower middle-class residents (including police and army) who travelled out to work, creating a substantial daily population 'swap'. Class distribution also undermined diversity in local social mixing (with middle-class having a preference for socialising outside of their community). Overall, the social permeability of these four higher efficiency communities contrasted with the lower transmission efficiency seen in the most socially closed communities, with far fewer 'outsiders' and less extra-community travel. Widespread participation in farming and/or fishing (across age and gender) seemed to correspond with only lower levels of transmission efficiency, while partial or no time spent farming/fishing corresponded with both high and low transmission efficiency. There was no pattern across indicators of wider participation in indoor activities, however, where only children watched video shows, transmission intensity appeared lower, with clear exceptions of communities 2 and 3.

DISCUSSION

This interdisciplinary analysis suggests that certain communities may have been protected from *Mtb* transmission in unique and sociologically complex ways. Communities with lower measures of transmission efficiency included, but were not limited to: those in which fishing and/or farming were particularly dominant across gender and age; where public transport was more ventilated and less overloaded; where less crowding took place in public space; where fewer 'outsiders' were present; where only men mostly travelled out of their community; where there was negligible evidence of indoor recreational activity; and where population and housing density was lower. Links between some of these socio-spatial aspects and *Mtb* transmission risk have been shown, using different approaches, in other sub-Saharan settings. In Malawi, Odone et al. identified that TB was more

For peer review only

common amongst those working in the cash economy than those in subsistence economy [24], and studies in South Africa have highlighted the significance of confined public transport and gathering places, with emphasis on the implications for air hygiene and rebreathed air volumes. [25-27]

From a distinctly qualitative angle, our work begins to unveil and define how the sociological topography of a particular community could play an important role in shaping transmission efficiency. Two meta-observations are significant in this regard. The first observation relates to the relative vulnerability of the communities with more outwardly orientated and socio-economically diverse features in comparison to their more socially secluded and/or agriculturally focused counterparts. Certain communities exhibit a dual dynamic of crowding and 'social permeability', which could be providing increased opportunity for the importation of *Mtb* and local aerosol transmission in poorly ventilated spaces. 'Social permeability' not only reflects the presence of non-residents within a community; it also captures the reach of 'mixing' across age and gender, and patterns of locals travelling out. The latter provides additional opportunity for extra–community *Mtb* exposure and infection (or re-infection), outside of the residential community. It seems that this dual dynamic of spatial 'closeness' and social 'openness' is relevant to increased *Mtb* transmission across the communities.

The second observation relates to a distinction that needs to be made between what we could call 'structural' and 'routine' variables. By structural variables we refer to socio-economic factors that are to a certain extent beyond people's agency. For instance, communities that are more structurally dependent on exchange and interaction with outsiders could be more vulnerable to *Mtb* exposure. Routine variables refer to habits or patterns of behaviour which although related to socio-economic factors are not obviously reducible to them. Watching video shows is a good example; it is a form of routine leisure activity which carries a particularly high presumptive risk for *Mtb* transmission because of the darkened, poorly ventilated and contained space in which it is conducted. For health policy, our distinction between 'structural' and 'routine' variables is important because the latter would be more malleable to intervention. Whilst structural dependency on the outside world cannot be altered easily, people can be encouraged to change the way in which they conduct routine leisure activities; for instance, a concerted effort could be made to increase ventilation in these places. [28]

Our secondary analysis has limitations. Figure 1 is exploratory and hypothesis-generating, and measures of association must be interpreted with appropriate caution. Nonetheless, results do suggest tentative patterns with respect to some indicators worthy of attention in future work. The estimates of ARI and TB prevalence are of limited precision, and ARI estimates can depend strongly

For peer review only

on the method of TST interpretation. Empirical estimates [29] and modelling [13] suggest ARI estimates in children are lower than in adults. However, ARI-to-prevalence ratios should still hold validity for relative comparisons between communities, since age-related contact patterns did not vary substantially between the communities. [13] Furthermore, we emphasise that the analysis did not include all community features relevant to social-spatial engagement. Congregation linked to both schools and churches has been established as a transmission risk factor in similar high burden settings [30, 31], yet we were unable to conduct BBS at schools. Importantly, churches emerged as significant (and mostly crowded) congregate features, with widespread attendance presenting in similar ways across age, gender and time. This consistency, across all communities, meant that churches could not be linked to sociological variation. We are also aware that local mines and prisons may have an important impact on *Mtb* transmission risk in communities [32-36], however, we were unable to identify any significance in our data. Additionally, there was scant evidence of women or men regularly attending traditional ceremonies, which may require further consideration: It is possible that the observational BBS did not hold enough ethnographic depth to capture the pervasiveness of more privately guarded socio-spatial activities.

Findings are context specific and data were effectively cross-sectional. We focused on interactions within localised geographical boundaries, and did not capture interactions on the far side of extra-community travel. Furthermore, fieldwork was conducted at a particular time of year and observations took place during the daytime, which limits the generalisability of our observations accordingly – not only across but also within the study communities. We anticipate relevant socio-spatial features and their role in mediating TB transmission will vary between contexts. However the dual dynamic of spatial closeness (crowding) and social openness (permeability) may be relevant in other settings, with further investigations valuable. The addition of pathogen genomic data might help generate or eliminate hypotheses on transmission location or context, and recent research is showing how quantitative measures of geographic mobility could easily be applied to studies of social permeability. [37] Whole genome sequencing could clarify whether importation is more or less relevant in certain communities and highlight transmission chains.

CONCLUSION

Localised sociological distinctions offered an explanation of why certain communities might have been more (or less) vulnerable to *Mtb* transmission. Interactions between social permeability and crowding appear relevant to increased transmission efficiency, at least in these Zambian communities. A conceptual framework differentiating structural and routine elements of high-risk socio-spatial

For peer review only

features could provide a valuable foundation for the development of contextualised public health interventions. For example, some socio-spatial aspects carrying transmission risk are easily adjusted (e.g. increasing ventilation on transport or at video shows), whilst more structural aspects are less malleable (e.g. reliance on public transport). The significance of social permeability could be further explored by integrating community level typologies with genome sequencing techniques able to map strain introduction events and community transmission chains.

ACKNOWLEDGEMENTS

We extend thanks to the communities, all participants, the regulatory authorities, the Ministry of Health and Zambart staff in Zambia for their support and involvement via the CODA and ZAMSTAR studies. In particular, Levy Chilikwela provided invaluable assistance with qualitative data analysis. We are also grateful to Patrick Baert, Peter Godfrey-Faussett and Jean Hunleth for feedback on various versions of this paper. Additionally, comments and recommendations from the journal's reviewers during the peer review process helped sharpen our report.

Contributorship: Substantial contribution to conception and design of ZAMSTAR: HA, VB, KS. Substantial contribution to conception and design of CODA: HA, VB, PJD, RW**.** Substantial contributions to conception and design of secondary analysis: VB, PJD, BM, EJM, RW. Acquisition of study data: HA, VB, PJD, AS, KS. Analysis and interpretation of data: PJD, EJM. Drafting of article: EJM. Revising critically for intellectual content: HA, VB, PJD, BM, EJM, RW.

Funding: This secondary analysis received no specific grant from any funding agency in the public, commercial or not-for-profit sectors. However: HA, VB, PJD, EJM and RGW were funded by the Bill and Melinda Gates Foundation (Consortium to Respond Effectively to the AIDS/TB Epidemic: 19790.01). RGW is funded by the Wellcome Trust (218261/Z/19/Z), NIH (1R01AI147321-01), EDTCP (RIA208D-2505B), UK MRC (CCF17-7779 via SET Bloomsbury), ESRC (ES/P008011/1), BMGF (OPP1084276, OPP1135288 & INV-001754), and the WHO (2020/985800-0). PJD is supported by the UK Medical Research Council (MR/P022081/1). (This UK funded award is part of the EDCTP2 programme supported by the European Union.) Funders had no involvement in the design, collection, analysis or interpretation of the data, in writing the manuscript or in the decision to submit.

Ethics: Formal ethical approval was not required for this secondary analysis of existing data. Earlier approval for fieldwork was obtained through the CODA Study from the University of Zambia Biomedical Research Ethics Committee (007-10-04), and London School of Hygiene and Tropical

For peer review only

Medicine (LSHTM) Ethics Committee (A211 3008). The ZAMSTAR study was approved by Ethical Review Boards of the University of Zambia, Stellenbosch University and the London School of Hygiene and Tropical Medicine.

Data Availability: Qualitative data are available from the last author upon reasonable request. Quantitative ZAMSTAR data are available from the sixth author upon reasonable request. Extra CODA study data are at Zenodo. DOI: http://doi.org/10.5281/zenodo.3874675. Relevant license details have Creative Commons Attribution (https://creativecommons.org/licenses/by/4.0/legalcode).

Supplemental Material: This comprises 'Annual Risk of Infection by Socio-Spatial Variables' (S1) and 'Prevalence of Disease by Socio-Spatial Variables (S2).

Competing interests: None declared.

REFERENCES

1. Global tuberculosis report 2020. Geneva: World Health Organisation; 2020. Licence: CC BY-NC-SA 3.0 IGO. https://www.who.int/tb/publications/global_report. Accessed 22 October 2020.

2. Ayles H, Schaap A, Nota A, et al. Prevalence of Tuberculosis, HIV and Respiratory Symptoms in Two Zambian Communities: Implications for Tuberculosis Control in the Era of HIV. *PLoS One* 2009;4(5):e5602. DOI:10.1371/journal.pone.0005602.

3. Kapata N, Chanda-Kapata P, O'Grady J, et.al. Trends of Zambia's tuberculosis burden over the past two decades. *Tropical Medicine and International Health* 2011;16(11):1404-1409. https://doi.org/10.1111/j.1365-3156.2011.02849.x. Accessed 22 October 2020.

4. Chanda-Kapata P, Kapata N, Klinkenberg E, et al. The prevalence of HIV among adults with pulmonary TB at a population level in Zambia. *BMC Infect Dis* 2017;17(236). DOI: 10.1186/s12879-017-2345-5.

5. Coffman J, Chanda-Kapata P, Marais BJ, et al. Tuberculosis among older adults in Zambia: burden and characteristics among a neglected group. *BMC Public Health* 2017;17(804). DOI:10.1186/s12889-017-4836-0.

6. Cremers AL, Manon de Laat M, Kapata N, et al. Assessing the Consequences of Stigma for Tuberculosis Patients in Urban Zambia. *PLoS One* 2015;10(3):e0119861. DOI:10.1371/journal.pone.0119861.

7. Bond V, Nyblade L. The importance of addressing the unfolding TB-HIV stigma in high HIV prevalence settings. *J Community Appl Soc Psychol* 2006;16:452-461. DOI:10.1002/casp.893.

8. Chanda D, Gosnell DJ. The Impact of Tuberculosis on Zambia and the Zambian Nursing Workforce. *Online J Issues Nurs* 2006;11(1):4. DOI: 10.3912/OJIN.Vol11No01Man03.

9. Murray EJ, Bond VA, Marais BJ, et al. High levels of vulnerability and anticipated stigma reduce the impetus for tuberculosis diagnosis in Cape Town, South Africa. *Health Policy Plan* 2013;28(4):410-418. DOI:10.1093/heapol/czs072.

10. Uys P, Marais BJ, Johnstone-Robertson S, et al. Transmission Elasticity in Communities Hyperendemic for Tuberculosis. *Clin Infect Dis* 2011;52(12):1399-404. DOI:10.1093/cid/cir229.

11. Hermans S, Horsburgh CR Jr., Wood R. A Century of Tuberculosis Epidemiology in the Northern and Southern Hemisphere: The Differential Impact of Control Interventions. *PLoS ONE* 2015;10(8):e0135179. DOI:10.1371/journal.pone.0135179.

12. Ayles H, Muyoyeta M, Du Toit E, et al. Effect of household and community interventions on the burden of tuberculosis in southern Africa: the ZAMSTAR community-randomised trial. *Lancet* 2013;382(9899):1183-1194. DOI:10.1016/S0140-6736(13)61131-9.

13. Dodd PJ, Looker C, Plumb ID, et al. Age and Sex Specific Social Contact Patterns and Incidence of *Mycobacterium tuberculosis* Infection. *Am J Epidemiol* 2016;183(2):156-166. DOI: https://doi.org/10.1093/aje/kwv160.

For peer review only

14. Sismandis C, Moulton LH, Ayles H, et al. Restricted Randomisation of ZAMSTAR: a 2 X 2 factorial cluster randomised trial. *Clinical Trials* 2008;5:316-327. DOI:10.1177/1740774508094747.

15. Ayles H, Sismanidis C, Beyers N, et al. ZAMSTAR, The Zambia South Africa TB and HIV Reduction study: Design of a 2 × 2 factorial community randomized trial. *Trials* 2008;9(63). DOI: https://doi.org/10.1186/1745-6215-9-63.

16. Shanaube K, Sismanidis C, Ayles H, et al. Annual Risk of Tuberculous Infection Using Different Methods in Communities with a High Prevalence of TB and HIV in Zambia and South Africa. *PLoS ONE* 2009;4(11):e7749. DOI: https://doi.org/10.1371/journal.pone.0007749.

17. Bond V, Ngwenya F, Murray E, et al. Value and Limitations of Broad Brush Surveys Used in Community-Randomized Trials in Southern Africa. *Qual Health Res* 2019;29(5):700-718. DOI:10.1177/1049732318809940.

18. Bond V, Hoddinott G, Viljoen L et al. How 'place' matters for addressing the HIV epidemic: evidence from the HPTN 071 (PopART) cluster-randomised controlled trial in Zambia and South Africa. *Trials* 2021; *22*(1), 1-13.

19. Bond V. Terrains and Tuberculosis: The Model Applied in Urgent Public Health Settings. In: Wallman S, ed. The Capability of Places: Methods for Modelling Community Response to Intrusion and Change. London, UK: Pluto Press, 2011: 80-110.

20. Murray EJ. The Social Terrain of Endemic Tuberulosis in and around Cape Town [MPhil dissertation]. Faculty of Health Sciences, Stellenbosch University, South Africa; 2010. Online at: http://hdl.handle.net/10019.1/5387 . Accessed 22 October 2020.

21. Churchyard G, Kim P, Sarita Shah N et al. What We Know About Tuberculosis Transmission: An Overview, *The Journal of Infectious Diseases* 2017; 216 (6): S629–S635. Online at: https://doi.org/10.1093/infdis/jix362. Accessed 25 June 2021.

For peer review only

22. Van Leth F, Van der Werf MJ, Borgdorff MW. Prevalence of tuberculous infection and incidence of tuberculosis; a re-assessment of the Styblo rule. *Bull World Health Organ* 2008;86(1):20-6. DOI:10.2471/blt.06.037804.

23. Simwinga M. Exploring the role of community participation in research studies: the case of two Community Advisory Boards in Zambia [doctoral dissertation]. London School of Hygiene and Tropical Medicine, United Kingdom; 2015.

24. Odone A, Crampin AC, Mwinuka V, et al. Association between socioeconomic position and tuberculosis in a large population-based study in rural Malawi. *PLoS One* 2013;8(10):e77740. DOI:https://doi.org/10.1371/journal.pone.0077740.

25. Andrews JR, Morrow C, Wood R. Modeling the role of public transportation in sustaining tuberculosis transmission in South Africa. *Am J Epidemiol* 2013;177(6):556–561. DOI:https://doi.org/10.1093/aje/kws331.

26. Issarow C, Mulder N, Wood R. Environmental and social factors impacting on epidemic and endemic tuberculosis: a modelling analysis. *R.Soc.open sci.* 2018 Jan;5(1). DOI:http://doi.org/10.1098/rsos.170726.

27. Andrews JR, Morrow C, Walensky RP, et al. Integrating Social Contact and Environmental Data in Evaluating Tuberculosis Transmission in a South African Township. *J Infect Dis* 2014;210(4):597–603. DOI:https://doi.org/10.1093/infdis/jiu138.

28. Richardson ET, Morrow CD, Kalil DB, et al. Shared air: a renewed focus on ventilation for the prevention of tuberculosis transmission [published correction appears in PLoS One. 2014; 9(8): e106216. *PLoS One*. 2014 May;9(5):e96334. DOI:10.1371/journal.pone.0096334.

29. Middelkoop K, Bekker LG, Liang H et al. Force of tuberculosis infection among adolescents in a high HIV and TB prevalence community: a cross-sectional observation study. *BMC Infect Dis*. 2011; 11(156). https://doi.org/10.1186/1471-2334-11-156.

30. Mathema B, Andrews JR, Cohen T, et al. Drivers of Tuberculosis Transmission. *J Infect Dis* 2017;216(suppl_6)S644-S653. DOI:10.1093/infdis/jix354.

For peer review only

31. Wood R, Racow K, Bekker L-G et al. Indoor Social Networks in a South African Township: Potential Contribution of Location to Tuberculosis Transmission. PLoS ONE. 2012; 7(6): e39246. https://doi.org/10.1371/journal.pone.0039246.

32. Henostroza G, Topp SM, Hatwiinda S, et al. The High Burden of Tuberculosis (TB) and Human Immunodeficiency Virus (HIV) in a Large Zambian Prison: A Public Health Alert. *PLoS One* 2013 8(8): e67338. DOI: https://doi.org/10.1371/journal.pone.0067338.

33. O'Grady J, Hoelscher M, Atun R, et al. Tuberculosis in prisons in sub-Saharan Africa – the need for improved health services, surveillance and control. *Tuberculosis* 2011;91 (2):173-178.DOI: https://doi.org/10.1016/j.tube.2010.12.002.

34. Chanda-Kapata P, Osei-Afriyie D, Mwansa C, et al. Tuberculosis in the mines of Zambia: A case for intervention. *Asian Pacific Journal of Tropical Biomedicine* 2016; 6(9):803-807. DOI:https://doi.org/10.1016/j.apjtb.2016.06.015.

35. Stuckler D, Basu S, McKee M, et al. Mining and Risk of Tuberculosis in Sub-Saharan Africa. *Amer J Public Health* 2011;101: 524-530. DOI https://doi.org/10.2105/AJPH.2009.175646.

36. Godfrey-Faussett P, Sonnenberg P, Shearer SC et al. Tuberculosis control and molecular epidemiology in a South African gold-mining community. *Lancet* 2000; 356(9235):1066-1071. DOI: https://doi.org/10.1016/S0140-6736(00)02730-6.

37. Robsky KO, Isooba D, Nakasolya O, et al. Characterization of geographic mobility among participants in facility- and community-based tuberculosis case finding in urban Uganda. *PLoS One*. 2021;16(5):e0251806. doi:10.1371/journal.pone.025180/

For peer review only

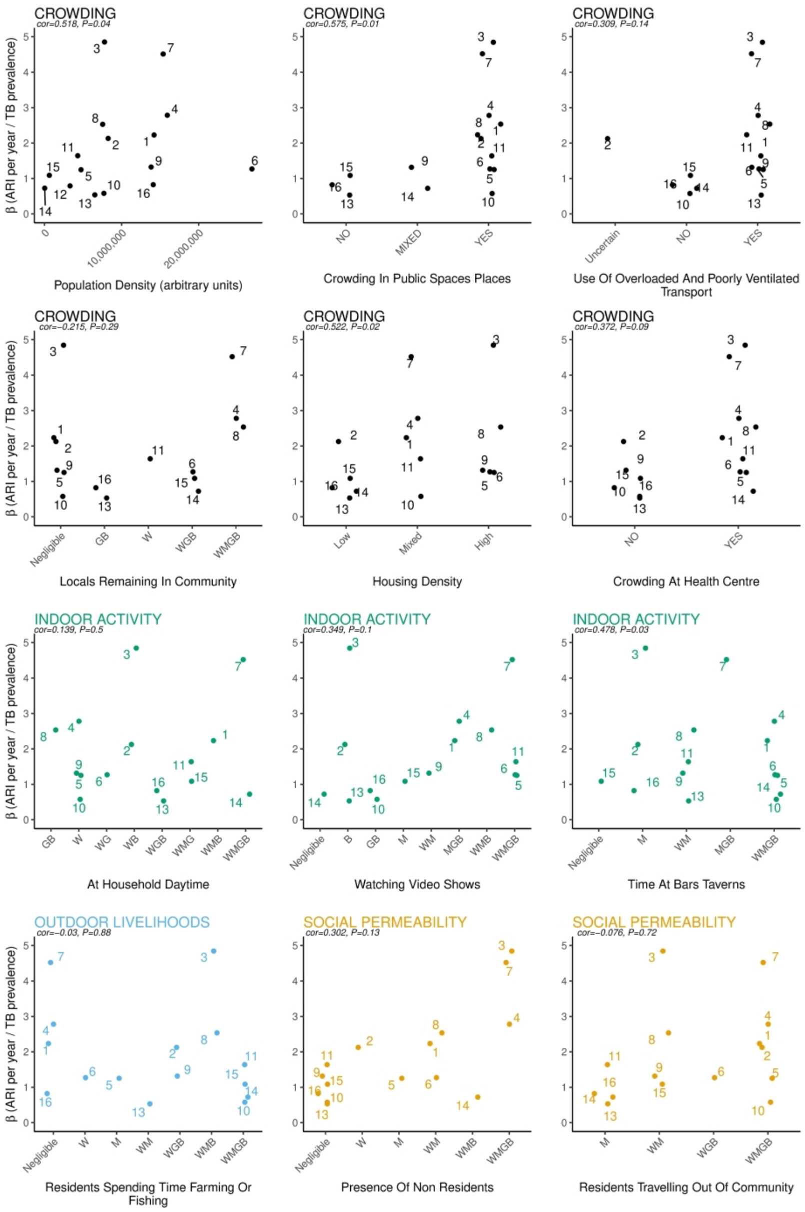

Figure 1: Transmission Efficiency (Ratio of Annual Risk of Infection to Disease Prevalence) by Socio-Spatial Variables

251x377mm (300 x 300 DPI)

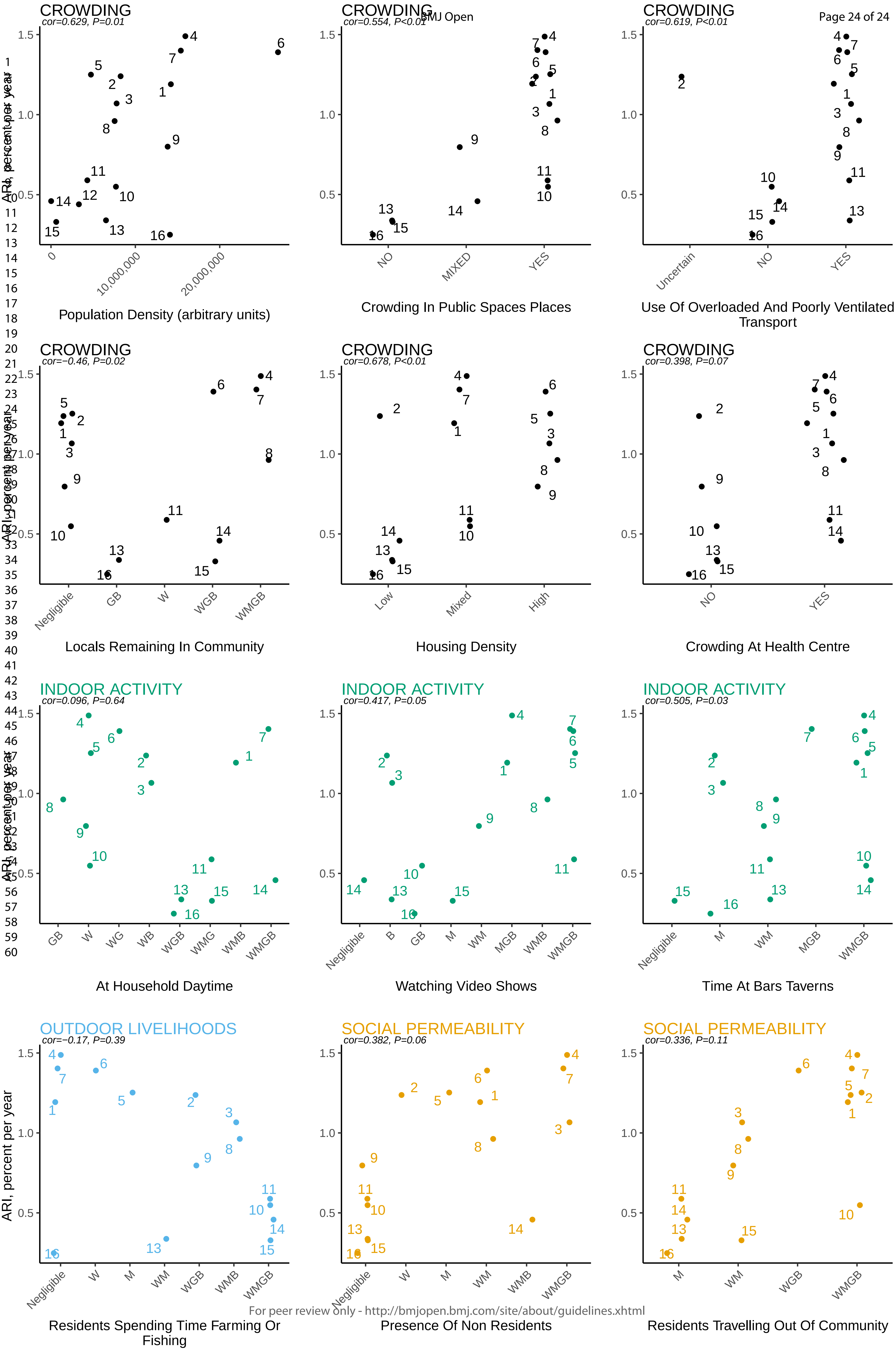
CROWDING
cor=0.629, P=0.01
Population Density (arbitrary units)
0
10,000,000
20,000,000
CROWDING
cor=0.554, P<0.01
Crowding In Public Spaces Places
NO
MIXED
YES
CROWDING
cor=0.619, P<0.01
Use Of Overloaded And Poorly Ventilated Transport
Uncertain
NO
YES
CROWDING
cor=−0.46, P=0.02
Locals Remaining In Community
Negligible
GB
W
WGB
WMGB
CROWDING
cor=0.678, P<0.01
Housing Density
Low
Mixed
High
CROWDING
cor=0.398, P=0.07
Crowding At Health Centre
NO
YES
INDOOR ACTIVITY
cor=0.096, P=0.64
At Household Daytime
GB
W
WG
WB
WGB
WMG
WMB
WMGB
INDOOR ACTIVITY
cor=0.417, P=0.05
Watching Video Shows
Negligible
B
GB
M
WM
MGB
WMB
WMGB
INDOOR ACTIVITY
cor=0.505, P=0.03
Time At Bars Taverns
Negligible
M
WM
MGB
WMGB
OUTDOOR LIVELIHOODS
cor=−0.17, P=0.39
Residents Spending Time Farming Or Fishing
Negligible
W
M
WM
WGB
WMB
WMGB
SOCIAL PERMEABILITY
cor=0.382, P=0.06
Presence Of Non Residents
Negligible
W
M
WM
WMB
WMGB
SOCIAL PERMEABILITY
cor=0.336, P=0.11
Residents Travelling Out Of Community
M
WM
WGB
WMGB
ARI, percent per year

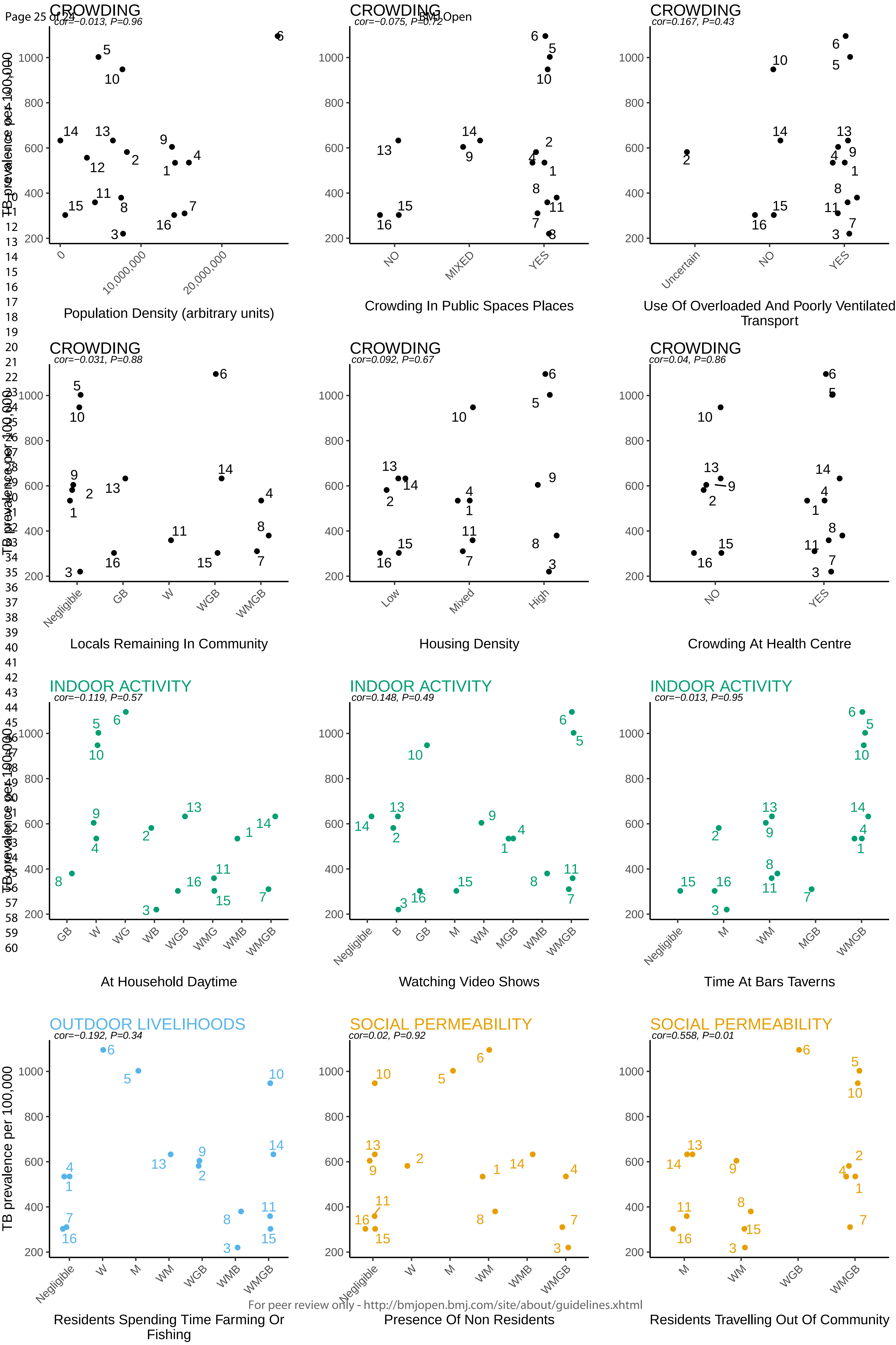
CROWDING
cor=−0.013, P=0.96
TB prevalence per 100,000
Population Density (arbitrary units)
0
10,000,000
20,000,000
CROWDING
cor=−0.075, P=0.72
Crowding In Public Spaces Places
NO
MIXED
YES
CROWDING
cor=0.167, P=0.43
Use Of Overloaded And Poorly Ventilated Transport
Uncertain
NO
YES
CROWDING
cor=−0.031, P=0.88
Locals Remaining In Community
Negligible
GB
W
WGB
WMGB
CROWDING
cor=0.092, P=0.67
Housing Density
Low
Mixed
High
CROWDING
cor=0.04, P=0.86
Crowding At Health Centre
NO
YES
INDOOR ACTIVITY
cor=−0.119, P=0.57
At Household Daytime
GB
W
WG
WB
WGB
WMG
WMB
WMGB
INDOOR ACTIVITY
cor=0.148, P=0.49
Watching Video Shows
Negligible
B
GB
M
WM
MGB
WMB
WMGB
INDOOR ACTIVITY
cor=−0.013, P=0.95
Time At Bars Taverns
Negligible
M
WM
MGB
WMGB
OUTDOOR LIVELIHOODS
cor=−0.192, P=0.34
Residents Spending Time Farming Or Fishing
Negligible
W
M
WM
WGB
WMB
WMGB
SOCIAL PERMEABILITY
cor=0.02, P=0.92
Presence Of Non Residents
Negligible
W
M
WM
WMB
WMGB
SOCIAL PERMEABILITY
cor=0.558, P=0.01
Residents Travelling Out Of Community
M
WM
WGB
WMGB
